# Loss of the PDLIM2 protein during chronic colitis promotes inflammation, impaired epithelium recovery, alterations to the microbiome and oxidative stress

**DOI:** 10.3389/fendo.2025.1720162

**Published:** 2026-02-10

**Authors:** Stephanie Ward, Orla T. Cox, Sara Roggiani, Tadgh Crowley, Silvia Turroni, Silvia Melgar, Rosemary O’Connor

**Affiliations:** 1 Cell Biology Laboratory, School of Biochemistry and Cell Biology, University College Cork, Cork, Ireland; 2 Department of Pharmacy and Biotechnology, University of Bologna, Bologna, Italy; 3 Department of Medical and Surgical Sciences, University of Bologna, Bologna, Italy; 4 APC Microbiome Ireland, University College Cork, Cork, Ireland

**Keywords:** colitis, oxidative stress, gut epithelium homeostasis, microbiome, integrin signalling, PDLIM2

## Abstract

**Introduction:**

Ulcerative colitis (UC) involves impaired wound healing processes contributing to sustained immune and microbial interactions that aggravate intestinal injury and may progress to colitis-associated cancer (CAC). Here we investigated whether PDLIM2, a known regulator of both epithelial and immune cell fate, contributes to colitis progression.

**Methods:**

PDLIM2 knockout mice (-/-) and wildtype littermates (+/+) were assessed for responses to dextran sodium sulphate (DSS)-induced colitis, and to aoxymethane +DSS. Microbiota were assessed using 16s rRNA amplicon sequencing. Mechanistic studies were carried out in Caco-2 cell cultures, and in silico analysis was carried out on single cell RNA sequencing data from patients with Ulcerative colitis or Crohn’s disease.

**Results and discussion:**

Compared to PDLIM2 +/+ mice, PDLIM2 -/- mice exhibited exacerbated and unresolved epithelial damage and inflammation accompanied by immune cell infiltration, which was precluded sufficient time to observe tunour development. PDLIM2 -/- mice exhibited altered basal gut microbial diversity, composition and predicted functionality compared to +/+ mice. Interestingly, in +/+ mice, PDLIM2 expression was lost over the course of DSS-induced colitis. Mechanistic studies in Caco-2 enterocyte cell cultures demonstrated that PDLIM2 suppression resulted in impaired cell adhesion signalling and sustained oxidative stress. In silico analysis of single cell RNA seq data sets from patients with ulcerative colitis and Crohn’s disease demonstrated that although PDLIM2 was clearly expressed in normal human colonic epithelial enterocyte populations, its expression declined in both ulcerative colitis and Crohn’s disease. We conclude that PDLIM2 is necessary for intestinal homeostasis through regulation of cell adhesion and antioxidant pathways, while loss of PDLIM2 sustains inflammation and epithelial damage.

## Introduction

The PDZ and LIM domain protein 2 (PDLIM2), also known as Mystique or SLIM, functions both to maintain epithelial cell polarity and regulate the differentiation and function of lymphocytes and macrophages (1–7). PDLIM2 expression is repressed in many cancers including colorectal cancer (CRC), breast, and lung carcinoma but is also highly expressed in aggressive cancers that have undergone epithelial-mesenchymal transition such as Triple Negative Breast Cancer (TNBC), oesophageal and prostate cancers (1–4). In epithelial cells PDLIM2 is necessary for adhesion, polarization and wound healing (4–6). In haemopoietic cells, PDLIM2 is required for macrophage adhesion and to enable full differentiation to the anti-inflammatory M2-like macrophage phenotype (7, 8), while it supports the differentiation of T-helper (Th) cell subsets (9, 10). A number of studies suggest that suppression of PDLIM2 in the epithelium and in immune cell populations contributes to inflammatory conditions such as autoimmune encephalitis (10), non-alcoholic fatty liver disease (NAFLD) (11), nephropathy (12), while PDLIM2 suppression also occurs during viral and bacterial infections (12, 13).

The functions of PDLIM2 in determining cell fate and in supporting either tumor suppression or cancer progression can be attributed to its actions as a cytoskeleton to nucleus courier protein. PDLIM2 location and stability in epithelial cells can be regulated by growth factor and adhesion signalling (3). It acts in the nucleus to regulate the protein stability and activity of key transcription factors, including signal transducer and activation of transcriptions (STATs), the p65 subunit of NF-κB, and β-catenin (3, 9, 14, 15). In this way it limits immune responses, while in epithelial cells it acts as a feedback regulator of the adhesion signalling responses through the β1-integrin/RhoA signalling axis (5). Although it is not fully understood how PDLIM2 expression and cytoskeleton or nuclear location is regulated, its repression and the genesis of lung cancer has been linked to cell non autonomous signalling events involving alveolar macrophage-derived reactive oxygen species (2, 8).

Considering its potential to regulate both epithelial and immune cell fate, we hypothesized that PDLIM2 activity may be implicated in the progression of inflammatory bowel disease (IBD), such as ulcerative colitis (UC). Patients with IBD carry an increased risk for developing colitis-associated CRC (CAC), depending on the overall severity, extent, and duration of inflammation that occurs in IBD (16–18). Disturbances to the integrity of the intestinal barrier are a key pathogenic feature observed in IBD (19). Under homeostatic conditions, an intestinal epithelial cell monolayer maintains gut homeostasis by acting as a selective barrier between the luminal content and underlying immune system. It integrates signals from the host epithelium and immune system with those originating from luminal microbiota, food-borne antigens, and toxins (20). However, it is not well understood how the maintenance of epithelial architecture constrains the activity of the immune response in IBD or its progression to cancer.

To investigate whether PDLIM2 contributes to intestinal epithelial maintenance and its role in intestinal inflammation, we used a PDLIM2 knockout mouse model, in which colitis and CAC were induced. We found in the mouse model that PDLIM2 absence had a profound effect on the severity of DSS-induced colitis, the integrity of the epithelium, and the resolution of inflammation compared to controls. Moreover, PDLIM2 became repressed in PDLIM2 +/+ mice treated with DSS. Interestingly, PDLIM2 -/- mice also demonstrated altered gut microbiota profiles at basal levels and in response to DSS challenge compared to their wildtype counterparts. Suppression of PDLIM2 in the enterocyte Caco-2 cell line resulted in dysregulated adhesion signalling and impaired antioxidant responses downstream of adhesion signals. Importantly, in human tissues atlases derived from patients with IBD (21, 22), we observed that PDLIM2 was suppressed during the progression of colitis. We conclude that PDLIM2 suppression during mouse and human colitis disrupts the architecture of the epithelial cell layer as well as anti-oxidant stress response pathways that are necessary for wound healing and suppression of inflammation.

## Results

### PDLIM2 -/- mice are more susceptible to DSS-induced colitis than PDLIM2 +/+ mice

**Figure 1:**
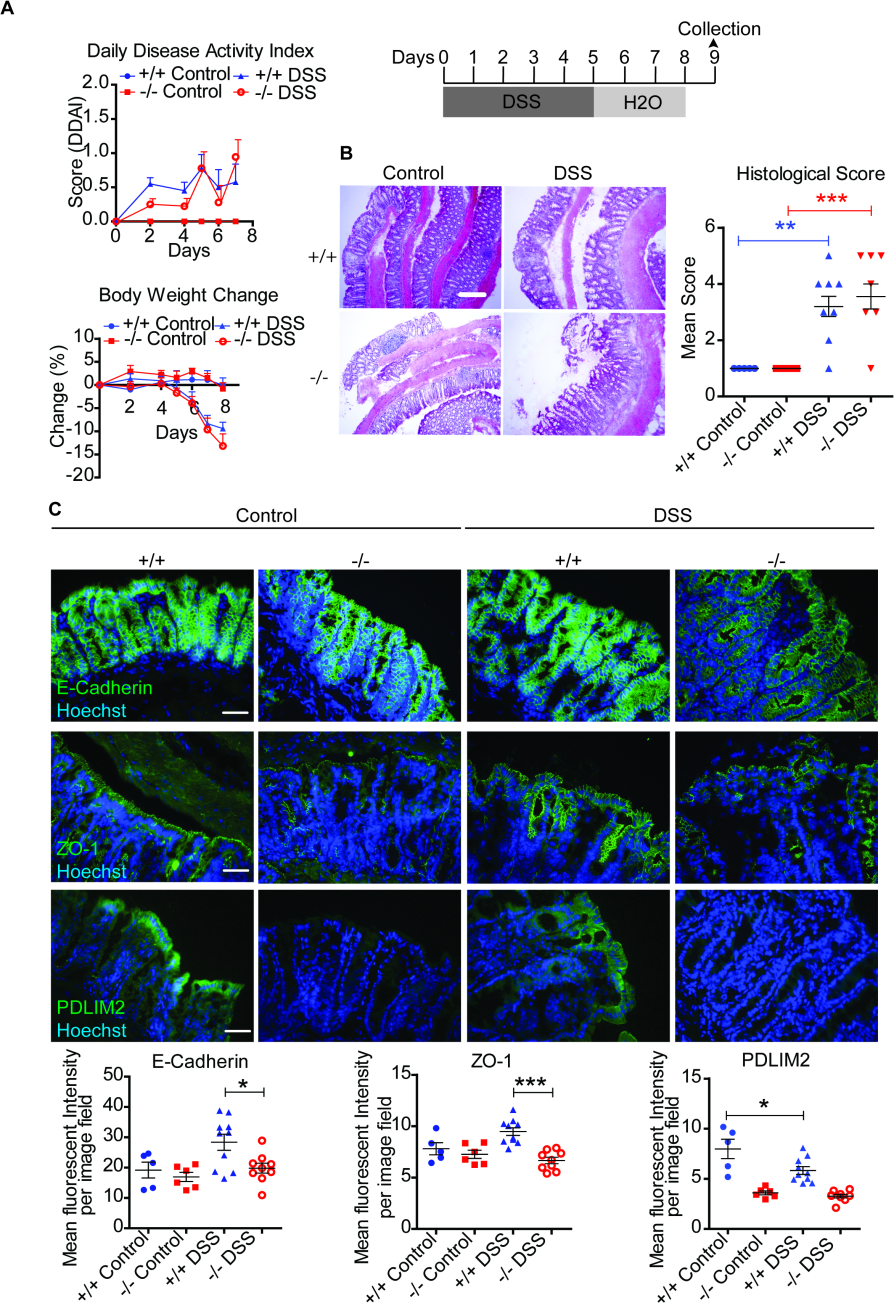
PDLIM2 +/+ and PDLIM2 -/- mice exhibit response to DSS-induced acute colitis Mice were administered 2.5% DSS dissolved in drinking water for a total of 5 days and followed by 3 days of normal drinking water. **(A)** Daily disease activity index (DDAI) incorporating stool consistency, fur texture, animal posture, and body weight change was assessed at 2 days intervals up to day 4, and daily to the end of the study. Data represents mean DDAI per group ± SD. Daily body weight change in mice was calculated by dividing the weight taken each day by the starting body weight at day 0 of each mouse and expressed as a percentage. Data represents the mean ± SD body weight change of mice per group each day. **(B)** H+E staining and histological scoring of distal colon tissues. Images were acquired at 10x magnification, with scale bars representing 200 µm. A representative image, and histological scoring was calculated from analyzing a minimum of 7 fields of stained tissue by assessing the degree of immune infiltration and architectural loss observed. **(C)** Immunofluorescence staining of distal colon sections for the adherens junctional marker, E-Cadherin, ZO-1 to assess tight junction integrity, and PDLIM2. Images were acquired at 40x magnification with scale bars representing 50µm. Scatter plots were generated by quantifying the mean fluorescence intensity per image field **(B)**. For B and C: Statistical analysis between groups was calculated by one-way ANOVA with Bonferroni *post-hoc* analysis. Asterisks (*) represent significant values where * *P* < 0.05, ** *P* < 0.01, and *** *P* < 0.001.

Our primary objective was to determine whether the presence or absence of PDLIM2 influences the progression of DSS- induced colitis or the potential for progression to CAC in a murine model. To address this, we followed two different experimental colitis schedules. The first involved short term exposure of PDLIM2 +/+ and -/- mice to DSS over 5 days and 3 days of regular water (**Figure 1A**). In this acute colitis setting, no differences were observed in the daily clinical symptoms between PDLIM2 +/+ and -/- mice. Histological analysis of harvested colonic tissues indicated similar levels of crypt disruption in DSS-treated PDLIM2 +/+ and -/- mice compared to normal crypts in untreated controls (**Figure 1B**). Immunostaining for the epithelial adherens junctional marker E-Cadherin and the tight junction marker, Zonula Occludens-1 (ZO-1) demonstrated that the cellular junctions were intact in DSS-treated PDLIM2 +/+ tissues and controls. However, in DSS-treated -/- mice tissues, E-Cadherin and ZO-1, were dispersed from cell adherens junctions to basal and apical surfaces (**Figure 1C**). Analysis of immune cell infiltration and markers of inflammation indicated similar levels of T cell and macrophage infiltration in PDLIM2 DSS-treated -/- and +/+ tissues, while levels of *NOS2* mRNA were increased although *CXCL10*, *IL-6*, *TNFa*, and *IFNg* were similar in DSS-treated -/- and +/+ mouse tissues (**Supplementary Figure 1**). Interestingly, PDLIM2 staining, which was observed at high levels along the distal tissue epithelial cell junctions in control PDLIM2 +/+ mouse tissue, was greatly reduced following DSS exposure (**Figure 1C**).

**Figure 2:**
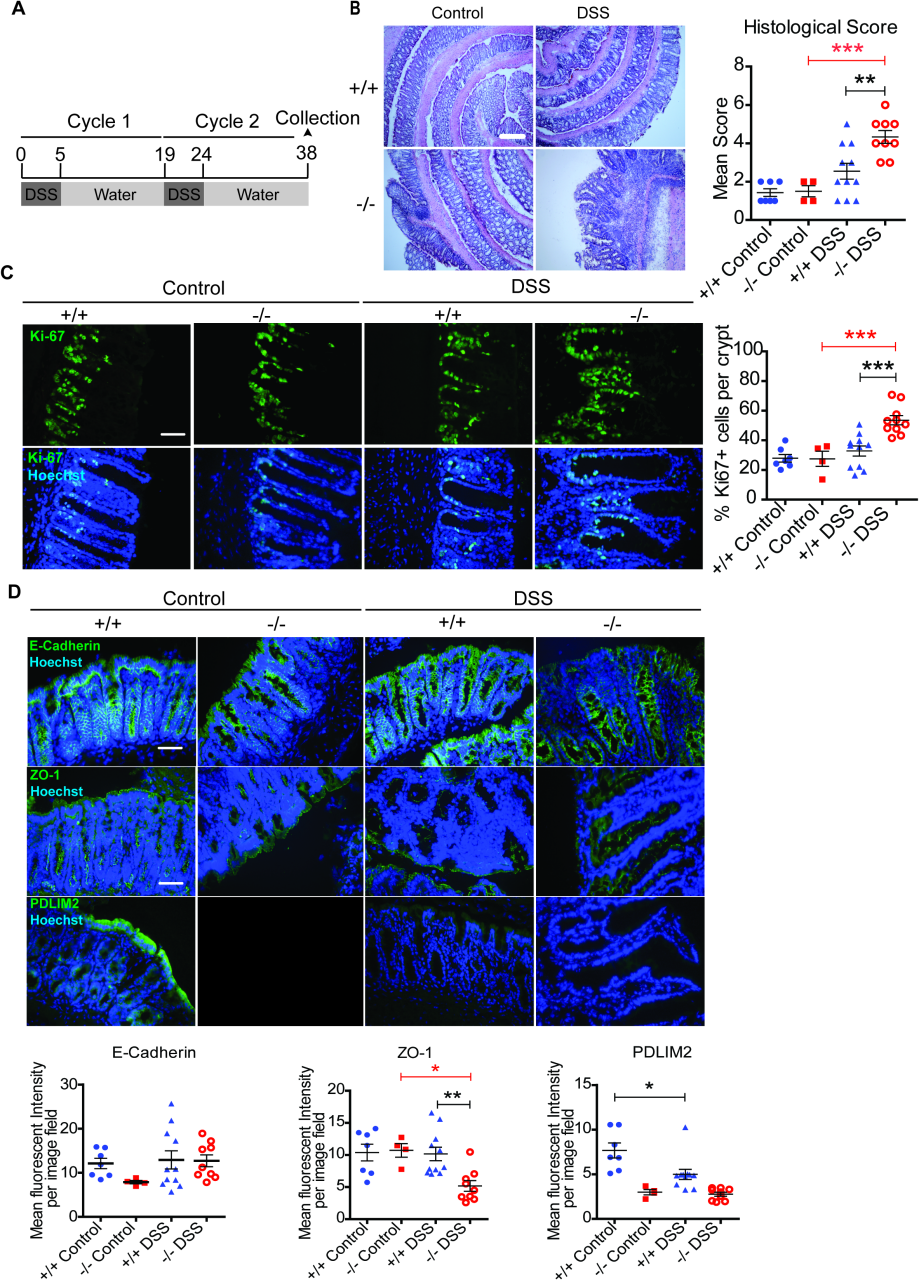
PDLIM2 -/- mice display exacerbated symptoms during chronic colitis. **(A)** Revised study protocol for PDLIM2 +/+ and PDLIM2 -/- mice with two cycles of DSS followed by 2 weeks of H_2_O (38 days) instead of three cycles of DSS, due to clinical symptoms (see **Supplementary Figure 1**). **(B)** Representative H+E staining of distal colon tissue from PDLIM2 +/+ and PDLIM2 -/- mice who received no treatment or 2x cycles of DSS. Original images were acquired at 10x magnification with scale bar representing 200µm. Histological scoring was calculated from H+E-stained tissue by assessing the degree of immune infiltration and architectural loss observed. **(C)** Immunofluorescence of Ki67 expression (green) and Hoechst nuclear staining (blue) within colon crypt of mice at 40x magnification. Scale bars represent 50µm. Data from scatter plot were generated by counting the number of Ki67+ cells within the colon crypts of mice from a minimum of 7 fields. Statistical analysis between groups was calculated by one-way ANOVA with Bonferroni *post-hoc* analysis. **(D)** Representative images of immunofluorescence staining for E-Cadherin, ZO-1, and PDLIM2 were used to calculate the mean fluorescence intensity within the distal colon tissue per image field. Images were acquired at 40x magnification and scale bars represent 50 µm. Statistical analysis between groups was calculated by one-way ANOVA with Bonferroni *post-hoc* analysis. For C and D the asterisk (*) represent significant values, where * *P* < 0.05, ** *P* < 0.01, and *** *P* < 0.001.

The second experimental setup investigated longer term exposure to DSS (chronic colitis) and potential progression to CAC. This involved AOM administration followed by three cycles of DSS or exposure to three cycles of DSS alone (**Supplementary Figure 1**, **Figure 2A**). Following daily monitoring, this study was halted on day 38 due to the severity of symptoms observed in PDLIM2 -/- mice that were exposed to two cycles of DSS alone, and their apparent failure to recover compared to PDLIM2 +/+ mouse cohorts (**Supplementary Figure 2**). Histological analysis of tissues from the DSS alone cohort demonstrated that epithelial barrier disruption was more evident in PDLIM2 -/- mice, than in +/+ mice following two cycles of DSS (**Figure 2B**). When we assessed the presence of proliferating cells in these tissues by immunofluorescence staining for Ki67, it was evident that higher levels of Ki67 staining was present in the epithelial crypts of PDLIM2 -/- compared to +/+ mice (**Figure 2C**), which indicated hyperplasia. However, there was no evidence of tumors in tissues from either AOM+DSS- or DSS alone-treated mice, which might be anticipated at this early stage in the protocol. The severity of disease progression in this model of chronic DSS-induced colitis does not allow sufficient time for tumors to develop. Therefore, tissues derived from the cohorts of mice that were treated with AOM+DSS mice were excluded from further analyses, and we focused on investigating the basis for the exacerbated chronic colitis observed in PDLIM2 -/- mice exposed to two cycles of DSS alone compared to their +/+ counterparts.

Epithelium integrity and the location of PDLIM2 was assessed by staining for E-Cadherin, ZO-1 and PDLIM2, respectively. E-Cadherin and ZO-1 staining indicated intact cellular junctions in DSS-treated PDLIM2 +/+ tissues and untreated controls. In contrast, colon tissue from DSS-treated PDLIM2 -/- mice displayed disrupted E-Cadherin and ZO-1 expression along the cell-cell junction and epithelial cell barrier (**Figure 2D**). Despite the evident crypt elongation in PDLIM2 -/- intestinal tissue, the levels of E-Cadherin were similar to control and DSS-treated PDLIM2 +/+ tissue. Notably, PDLIM2 expression levels were greatly reduced in PDLIM2 +/+ mice exposed to DSS alone (**Figure 2D**). This was similar to observations in mice exposed to one cycle of DSS (**Figure 1A**), and indicates that suppression of PDLIM2 persists in DSS-treated mice, despite the apparent recovery of the epithelial architecture (**Figure 2D**).

Taken together, the results demonstrate that PDLIM2 -/- mice fail to recover and are significantly more affected by chronic DSS-induced colitis than their +/+ cohorts. Moreover, the expression of PDLIM2 at the colon epithelial barrier becomes suppressed in response to either acute or chronic DSS-induced colitis, along with disrupted integrity of the epithelial barrier.

### Inflammatory environment persists in chronic DSS-treated PDLIM2 -/- mice

**Figure 3:**
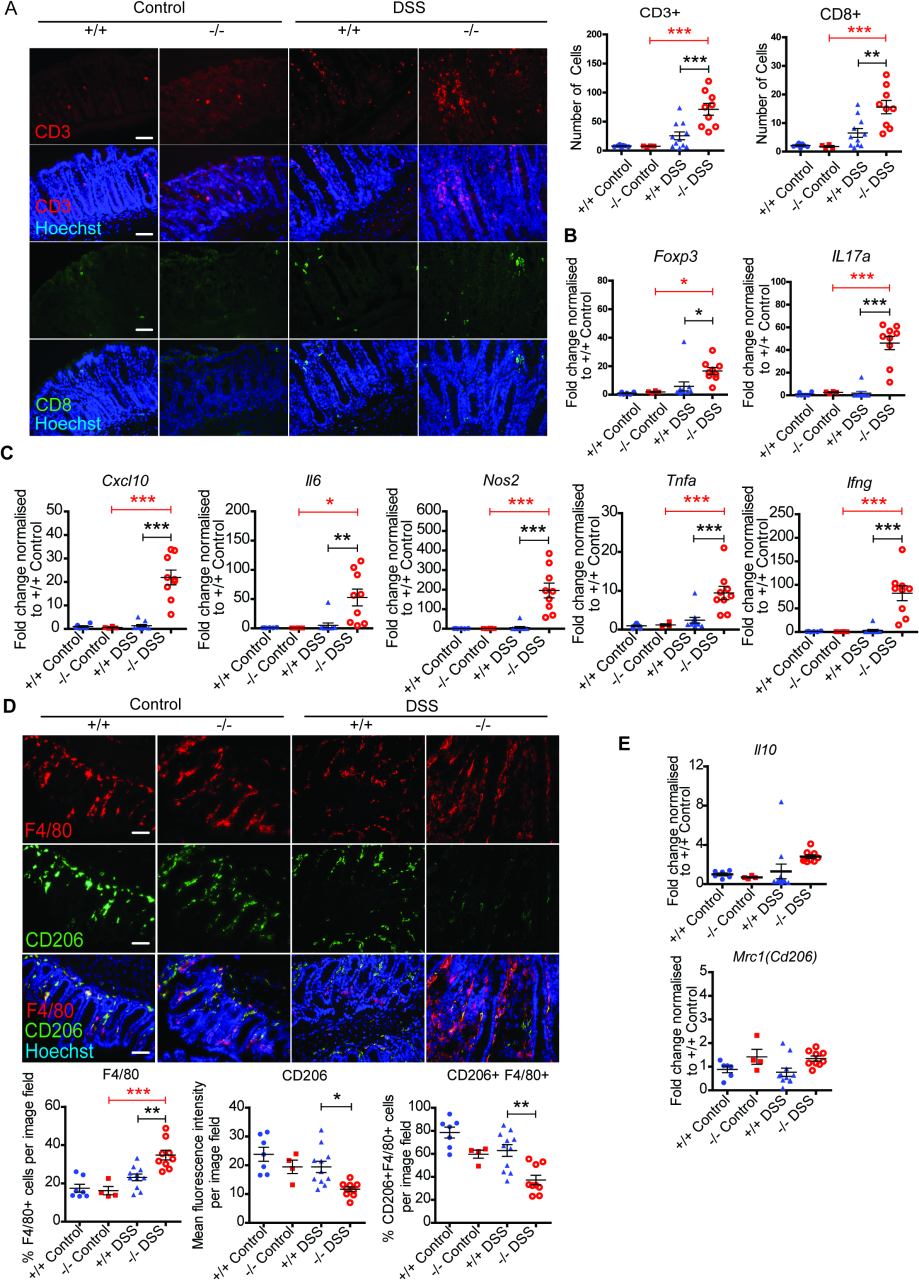
DSS-treated PDLIM2 -/- mice display greater colon inflammation than PDLIM2 +/+ mice. **(A)** Distal colon tissue from control and DSS-treated mice was stained with an anti-CD3 (red) and anti-CD8+ (green) antibody and Hoechst nuclear stain. Representative images acquired at 40x magnification are shown with scale bars representing 50µm. Quantitative analysis was conducted by counting the number of CD3+ and CD8+ cells per image field from a minimum of 7 fields **(B)**. Gene expression of *Foxp3* and *Il17a* were assessed by RT-qPCR of RNA extracted from distal colon tissue. **(C)** Expression levels of pro-inflammatory cytokines *Cxcl10*, *Il6*, *Ifng*, *Tnfa*, and *Nos2* were assessed by RT-qPCR analysis using RNA extracted from distal colon tissues. **(D)** Total macrophage expression and the prevalence of M2-like macrophages were assessed by staining tissues for-F4/80 (red), CD206 (green) with nuclei (Hoechst blue). Images were acquired at 40x magnification with scale bars representing 50µm **(A)**. Quantitative analysis was carried out by calculating the percentage of cells positive for F4/80, the level of CD206 expressed based on fluorescence intensity of signal per percentage of positive CD206 cells, and the percentage of CD206 positive cells as a fraction of total of F4/80 positive cells. **(E)** Gene expression was determined by RT-qPCR for *Mrc1* and *Il10*. All scatter plots were generated from mean ± SEM per group and significance calculated using one-way ANOVA and Bonferroni *post-hoc*. * *P* < 0.05, ** *P* < 0.01, and *** *P* < 0.001.

We next asked whether the dysplasia observed in tissues from DSS-treated PDLIM2 -/- mice was associated with surrounding inflammation and/or infiltration of macrophages that may be involved in wound healing. We first assessed the infiltration of T lymphocyte populations and expression of inflammatory cytokines in DSS-treated PDLIM2 -/- and +/+ mouse tissues. The overall numbers of T-lymphocytes (CD3+) and cytotoxic T-lymphocytes (CD8+) were low in control untreated -/- and +/+ mouse tissues (**Figure 3A**) and were not significantly different in PDLIM2 +/+ mice exposed to DSS. However, CD3+ and CD8+ lymphocytes were significantly elevated in colon tissue from PDLIM2 -/- mice that were exposed to DSS. This apparently more persistent infiltration of total lymphocytes and cytotoxic T cells may reflect a stronger immune response or, alternatively, reduced resolution of the immune response in the PDLIM2 -/- mice experiencing chronic colitis.

Levels of *Foxp3* and *IL17a* mRNA in tissues were assessed by qPCR, as markers of regulatory T cells and Th 17 cell infiltration. There was no significant difference in the expression of either *Foxp3* or *IL17a* mRNA between control mice and PDLIM2 +/+ mice exposed to DSS (**Figure 3B**). However, significantly higher levels of both *Foxp3* and *IL17a* mRNA were observed in DSS-treated PDLIM2 -/- tissues than in PDLIM2 +/+ tissues or untreated controls. Expression levels of *CXCL10*, *IL-6*, *TNFa*, *IFNg* and *NOS2* mRNA were all significantly higher in DSS-treated PDLIM2 -/- mice tissue than in DSS-treated PDLIM2 +/+ and untreated controls (**Figure 3C**).

Macrophages were detected by F4/80 staining. A higher staining intensity was observed in colon tissues from DSS-treated PDLIM2 -/- mice, compared to PDLIM2 +/+ mice or control mice, indicating more infiltration of macrophages in the presence of chronic inflammation (**Figure 3D**). CD206 staining was used to assess the presence of wound healing macrophages. Levels of CD206 expression relative to F4/80 expression were lower in tissue from PDLIM2 -/- mice compared to DSS-treated PDLIM2 +/+ mice, while fewer macrophages were observed to co-express both F4/80 and CD206 in tissue from DSS-treated PDLIM2 -/- mice (**Figure 3D**). Lower CD206 expression suggests that the recruitment and/or differentiation of wound-healing macrophages is impaired in PDLIM2 -/- mice. This is consistent with our previous findings (7) and also with the observation that levels of mRNA expression for *CD206* and *IL10* mRNA were similar in PDLIM2 -/- and PDLIM2 +/+ tissue, despite the surrounding inflammation (**Figure 3E**).

Taken together the expression profile of, T cells, cytokines, *NOS2* and macrophages demonstrate that a highly inflammatory environment persists following DSS-induced chronic colitis in PDLIM2 -/- mice.

### Loss of PDLIM2 alters gut microbiota composition and functionality

Given the observed apparently inherent sensitivity of PDLIM2 -/- mice to epithelial disruption and inflammation, we next compared the gut microbiota profile in these mice to their PDLIM2 +/+ counterparts. Stool sampling was performed on the day of culling for a total of 22 samples (6 for untreated control PDLIM2 +/+ mice, 7 for DSS-treated PDLIM2 +/+, 4 for untreated PDLIM2 -/- and 5 for DSS-treated PDLIM2 -/-). Samples were processed by 16S rRNA amplicon sequencing, yielding 345,377 high-quality reads (mean ± SEM, 15,699 ± 2,546).

PDLIM2 +/+ control mice were distinguished from the other three groups by lower alpha diversity (Wilcoxon test, p≤0.030) (**Supplementary Figure 3A**). Similarly, PDLIM2 +/+ control mice segregated from the other groups in the weighted UniFrac-based PCoA (PERMANOVA, p<0.006) (**Supplementary Figure 3B**), suggesting that PDLIM2 absence and DSS treatment similarly affected the dominant fraction of the microbial ecosystem. In contrast, in the unweighted UniFrac-based PCoA, there were trends towards segregation between PDLIM2 +/+ control mice and PDLIM2 -/- control mice (p=0.062), PDLIM2 +/+ DSS mice and both PDLIM2 +/+ control and PDLIM2 -/- control mice (p≤0.088), and PDLIM2 -/- untreated mice and PDLIM2 -/- DSS-treated mice (p=0.053) (**Supplementary Figure 3B**), suggesting condition-specific effects on the subdominant microbiota fraction.

**Figure 4:**
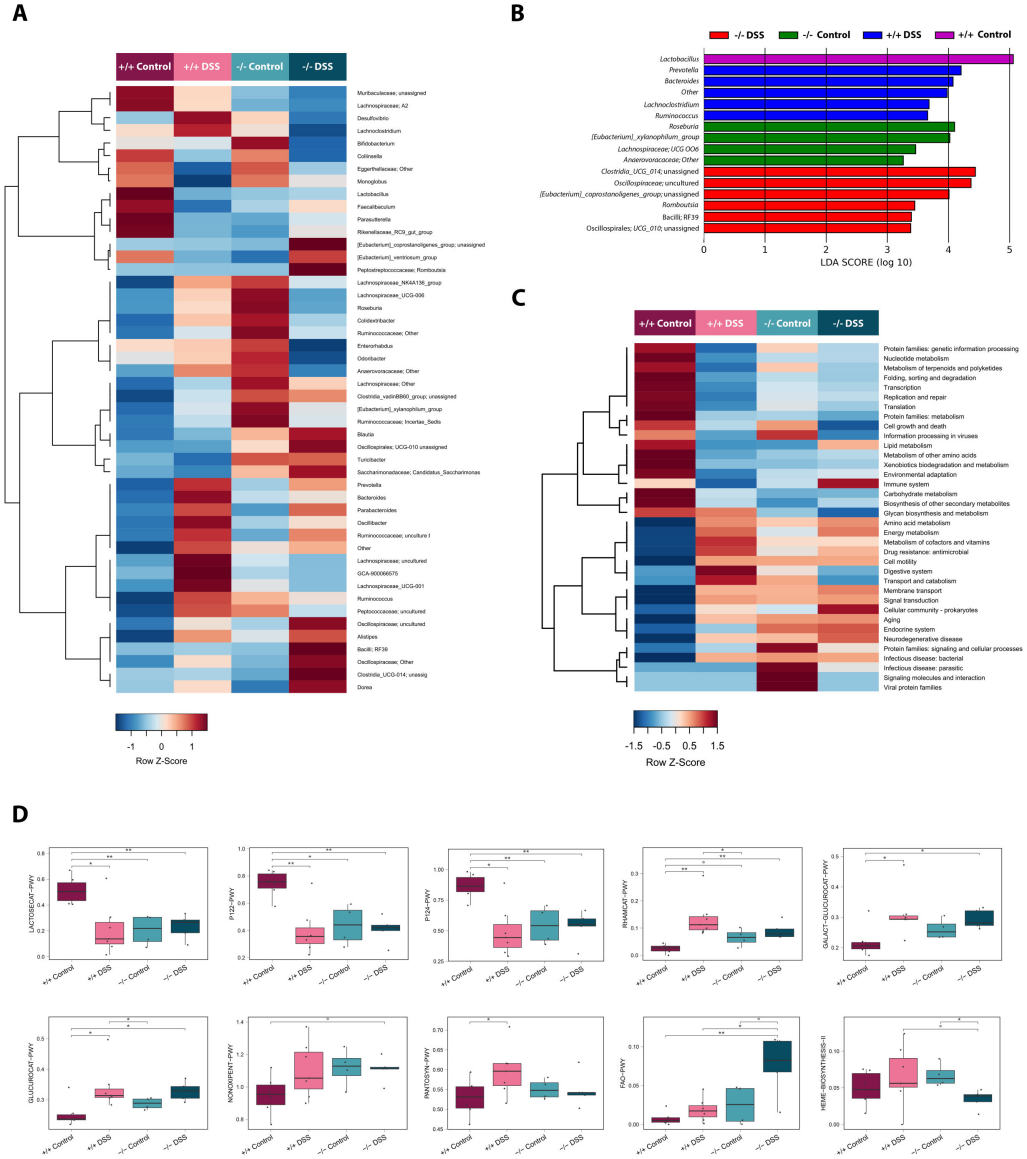
Loss of PDLIM2 alters gut microbiota composition and functionality. **(A)** Heatmap showing Ward-linkage clustering based on Spearman correlation coefficients of the mean relative abundance of gut microbiota genera from PDLIM2 +/+ mice treated with water (+/+ Control) or DSS (+/+ DSS) and PDLIM2 -/- mice treated with water (-/- Control) or DSS (-/- DSS). The relative Z score is reported. **(B)** Discriminating genera between groups were identified by linear discriminant analysis (LDA) effect size (LEfSe) analysis. **(C)** Heatmap showing Ward-linkage clustering based on Spearman correlation coefficients of the mean relative abundance of predicted KEGG pathways in the gut microbiota of the study groups. The relative Z score is reported. **(D)** Boxplots showing the relative abundance (%) distribution of predicted MetaCyc pathways differentially represented between groups. Wilcoxon test, * p ≤ 0.05; ** p ≤ 0.01; ° p ≤ 0.09. LACTOSECAT-PWY: lactose degradation pathway; P122-PWY: heterolactic fermentation pathway; P124-PWY: *Bifidobacterium* shunt; RHAMCAT: rhamnose degradation pathway; GALACT-GLUCUROCAT-PWY: superpathway of hexuronide and hexuronate degradation pathway; GLUCUROCAT-PWY: superpathway of β-D-glucuronosides degradation; NONOXIPENT-PWY: pentose phosphate pathway (non-oxidative branch) I; PANTOSYN-PWY: superpathway of coenzyme A biosynthesis I; HEME-BIOSYNTHESIS-II: heme group biosynthesis pathway; FAO-PWY: fatty acid β-oxidation I pathway. See also **Supplementary Figures 2, 3, 4 and 5**.

From a taxonomic standpoint, the most represented phyla were Bacteroidota (mean relative abundance in the whole cohort ± standard error of the mean, 47.3% ± 2.6%) and Firmicutes (46% ± 3.0%), followed by Actinobacteriota (3.8% ± 0.6%). The latter decreased in PDLIM2 -/- DSS mice compared to PDLIM2 -/- and PDLIM2 +/+ control mice (Wilcoxon test, p ≤ 0.051) (**Supplementary Figure 4A** and **Supplementary Table 1**). At the family level, the microbial ecosystem of all groups was dominated by *Muribaculaceae* (42.2% ± 2.3%), *Lactobacillaceae* (19.4% ± 2.6%), *Lachnospiraceae* (15.4% ± 2.6%) and *Oscillospiraceae* (3.5% ± 0.7%). However, PDLIM2 +/+ control mice differed from the other groups by higher proportions of *Lactobacillaceae* (p ≤ 0.022), and lower proportions of *Oscillospiraceae* (p≤0.025), *Bacteroidaceae* (p ≤0.034), *Prevotellaceae* (p≤0.088) and Clostridia_vadinBB60_group (p≤0.090) (**Supplementary Figure 4B** and **Supplementary Table 1**). On the other hand, DSS-treated PDLIM2 -/- mice were distinguished from all other groups by higher proportions of *Peptostreptococcaceae* (p ≤ 0.044), *[Eubacterium]_coprostanoligenes*_group (p ≤ 0.073), Clostridia_UCG-014 (p ≤0.052) and Firmicutes; RF39 (p ≤ 0.073). PDLIM2 -/- DSS-treated mice also had higher proportions of *Erysipelotrichaceae* (p =0.014) and *Clostridiaceae* (p=0.089) compared to DSS-treated PDLIM2 +/+ mice, and higher proportions of *Christensenellaceae* (p =0.043) and lower proportions of *Bifidobacteriaceae* (p =0.015) compared to PDLIM2 -/- control mice. Independent of DSS treatment, the absence of PDLIM2 resulted in a trend of increase in *Lachnospiraceae* (p ≤0.052) and *Tannerellacaeae* (p ≤0.09) compared to PDLIM2 +/+ control mice. Finally, both PDLIM2 -/- control mice and DSS-treated PDLIM2 +/+ mice showed higher proportions of *Ruminococcaceae* (p ≤0.025) and *Peptococcaceae* (p ≤0.05) compared to PDLIM2 +/+ control mice. Consistent results were obtained at the genus level (**Figure 4A** and **Supplementary Figure 4C**). In particular, according to LefSe analysis, the main discriminating genera were: i) *Lactobacillus* for PDLIM2 +/+ control mice; ii) *Prevotella*, *Bacteroides*, *Ruminococcus* and *Lachnoclostridium* for PDLIM2 +/+ DSS mice; iii) *Roseburia, [Eubacterium]_xylanophilum_*group, *Lachnospiraceae*;UCG 006 and *Anaerovoracaceae*;other for PDLIM2 -/- control mice; and iv) Clostridia_UCG_014;unassigned, *Oscillospiraceae*;uncultured, *[Eubacterium]_coprostanoligenes*_group;unassigned, *Rombutsia*, Bacilli; RF39, and Oscillospirales; UCG-010;unassigned for PDLIM2 -/- DSS mice (**Figure 4B** and **Supplementary Figure 5**).

Regarding inferred metagenomics (**Figure 4C**, **Supplementary Figure 6** and **Supplementary Table 1**), PDLIM2 +/+ control mice showed the highest levels of a number of central metabolic pathways, including nucleotide metabolism, replication and repair, genetic information processing, transcription and translation, protein metabolism, metabolism of other amino acids, carbohydrate metabolism, biosynthesis of secondary metabolites, xenobiotics biodegradation and metabolism, and environmental adaptation (p ≤ 0.066). On the other hand, DSS-treated PDLIM2 +/+, PDLIM2 -/- control, and DSS-treated PDLIM2 -/- mice shared enrichment in pathways associated with energy metabolism, amino acid metabolism, metabolism of cofactors and vitamins, bacterial infectious diseases, antimicrobial resistance, signal transduction, and cell motility (p ≤ 0.051). Independent of DSS treatment, the absence of PDLIM2 resulted in an increase in microbial pathways related to the endocrine system compared to PDLIM2 +/+ control and DSS-treated PDLIM2 +/+ mice (p ≤ 0.01). PDLIM2 -/- control mice also showed enrichment in pathways associated with parasitic infectious diseases compared to DSS-treated PDLIM2 -/- and PDLIM2 +/+ mice (p ≤ 0.063). Finally, DSS-treated PDLIM2 -/- mice showed higher levels of pathways related to the immune system compared to DSS-treated PDLIM2 +/+ mice, and pathways related to cellular community-prokaryotes compared to PDLIM2 +/+ control mice (p ≤ 0.048). In terms of MetaCyc pathways (**Figure 4D** and **Supplementary Table 1**), lactose degradation, heterolactic fermentation, and *Bifidobacterium* shunt were overrepresented in PDLIM2 +/+ control mice compared to all other groups (p ≤ 0.022), while rhamnose degradation was overrepresented in the other groups (p ≤ 0.067). DSS-treated PDLIM2 -/- mice were distinguished from all other groups by an enrichment in fatty acid β-oxidation I pathway (p ≤ 0.063) and showed an increase in pentose phosphate pathway (non-oxidative branch) I compared to PDLIM2 +/+ control mice (p =0.052). Superpathway of coenzyme A biosynthesis I was enriched in DSS-treated PDLIM2 +/+ mice compared to PDLIM2 +/+ control mice (p=0.035). Regardless of the presence of PDLIM2, DSS treatment resulted in enrichment in the superpathway of hexuronide and hexuronate degradation and superpathway of β-D-glucuronosides degradation compared to PDLIM2 +/+ control mice (p ≤ 0.035).

Overall, the data demonstrate that loss of PDLIM2 is sufficient to alter the diversity, composition and predicted functionality of the gut microbiota, and that DSS further alters the microbiota composition.

### PDLIM2 suppression in Caco-2 epithelial cells impairs adhesion signalling in the presence of oxidative stress

We next turned our attention to investigating the mechanism(s) through which lack of PDLIM2 affects the phenotype of intestinal epithelial cells, by suppressing its expression in, Caco-2 cells. The Caco-2 cell line is derived from a colorectal carcinoma and is extensively used for studies of intestinal epithelial cell function and the colonic epithelial barrier because they retain the ability to spontaneously differentiate into a monolayer of cells with features of absorptive enterocytes (23, 24). We first surveyed the expression of PDLIM2 in Caco-2 cells along with adhesion receptors E-Cadherin and β1-integrin, and compared with other CRC cells. As can be seen in supplementary **Supplementary Figure 5**, PDLIM2 is expressed in Caco-2 cells along with E-Cadherin, β-catenin and β1-integrin (ITGB1), confirming their epithelial phenotype.

**Figure 5:**
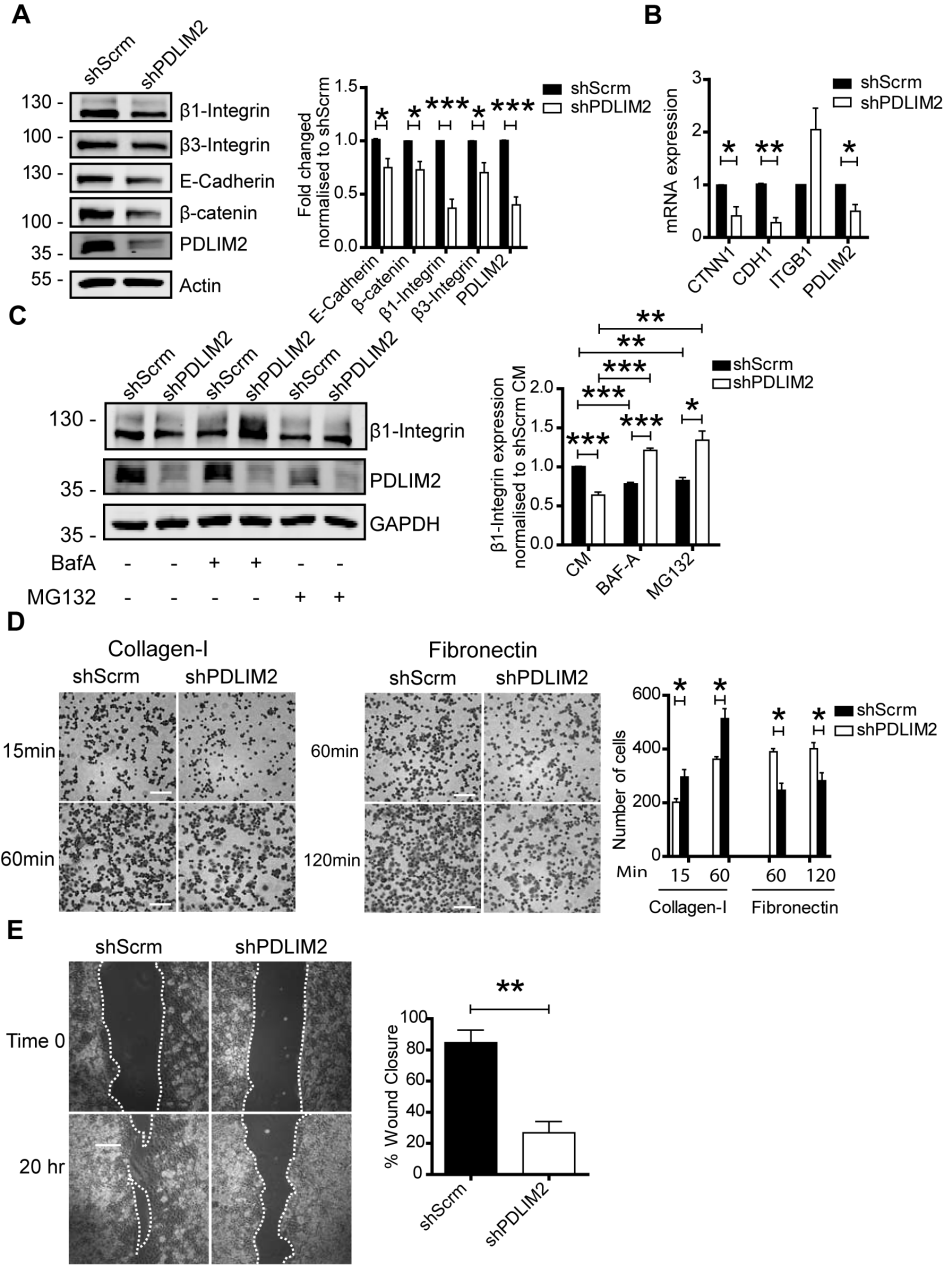
Suppression of PDLIM2 in Caco-2 cells impairs adhesion signalling. Caco-2 cell lines with PDLIM2 stably suppressed by shRNA (shPDLIM2) or cells expressing a control shRNA (shScrm) were selected. **(A)** Western blot showing expression levels of β1-Integrin, β3-Integrin, E-Cadherin, β-catenin, and PDLIM2 in these cells following 48 hours in culture. **(B)** RT-qPCR analysis of *CTNN1*, *CDH1*, *ITGB1*, and *PDLIM2* gene expression in shScrm and shPDLIM2 cells **(C)** Bafilomycin A (10nM) or MG132 (10µM) were included in cell cultures for 14 hours prior to cell lysis for analysis of PDLIM2, and β1-Integrin expression by western blotting **(D)**. Cell adhesion capacity determined in cells seeded onto Collagen-I-coated plates for 15 and 60 minutes, or fibronectin-coated plates for 60 and 120 minutes, respectively. Non-adherent cells were removed by washing with PBS, and adhered cells were fixed and stained with crystal violet before being photographed at 20x magnification. Crystal violet staining was quantified using Image J processing software. Scale bars represent 100µm. **(E)** Cell motility was assessed in wound closure assays using Ibidi chambers following 24 hours culture. The area of the wound was measured at time 0 and at 20 hr, with the percentage closure expressed relative to time 0. Scale bars of images refer to 100µm. All bar graphs represent the mean ± SEM of three independent experiments where p-values were calculated using the student t-test and represented as * = *P* < 0.05, ** = *P* < 0.01, and *** = *P* < 0.001.

Following stable suppression of PDLIM2 in Caco-2 cells (shPDLIM2), the expression of β1-integrin (ITGB1), β3-integrin, E-Cadherin, and β-catenin protein levels were all significantly lower than in shScrambled (shScrm) controls (**Figure 5A**). However, although ITGB1 protein levels were lower the mRNA levels were elevated (**Figure 5B**), and protein levels could be restored by inhibition of either lysosome or proteasome-mediated protein degradation with Bafilomycin A or MG132. This indicates that the turnover of β1-integrin is enhanced when PDLIM2 is suppressed, compared with shScrm control cells that exhibited low levels of β1-integrin protein turnover (**Figure 5C**). In line with the altered expression of adhesion molecules, the adhesion of shPDLIM2 cells to Collagen-1 and fibronectin was also impaired (**Figure 5D**) as well as their capacity for cell motility in wound healing assays (**Figure 5E**) and formation of acinar spheroids was also impaired (**Supplementary Figure 8**).

**Figure 6:**
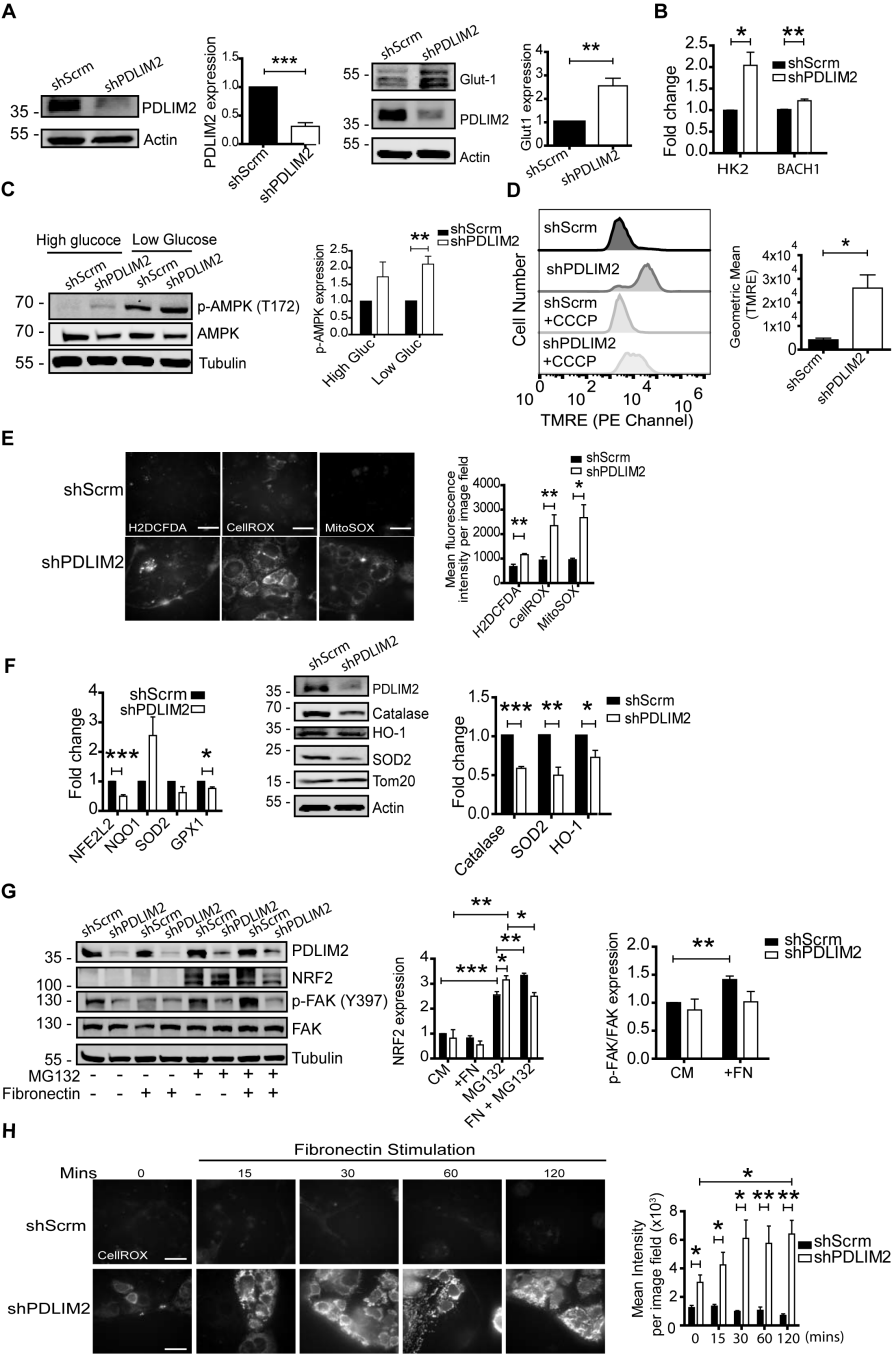
Persistent metabolic stress and impaired anti-oxidant buffering in Caco-2 cells with PDLIM2 suppressed. **(A)** Protein expression of PDLIM2 and Glut-1 in shScrm and shDPLIM2 Caco-2 cells that were cultured in complete medium. **(B)** Gene expression of *HK-2* and *BACH1* determined by qPCR using cDNA generated from mRNA isolated from shScrm and ShPDLIM2 Caco-2 cells. **(C)** Western blots showing p-AMPK (T172), AMPK, and Tubulin as a loading control in cell lysates derived from shScrm or shPDLIM2 Caco-2 cells that were cultured in either high or low glucose-containing medium for 48hrs. **(D)** Mitochondrial membrane potential was assessed by flow cytometry in adherent shScrm and shPDLIM2 cells by incubation in medium containing 500nM TMRE dye for 20 minutes, before detachment and analysis. In addition, Carbonyl cyanide m-chlorophenyl hydrazone (20µM CCCP) was added 20 minutes as a control for mitochondrial uncoupling (bottom two histograms). Data are presented as mean ± SEM of geomean fluorescence from three independent experiments. **(E)** Adherent cells were incubated with 10µM of H_2_DCFDA for 30 minutes in the dark to quantify hydrogen peroxide species, 30 minutes with 5µM of CellROX to quantify superoxide species, or 10 minutes with 500nM MitoSOX to quantify mitochondrial-specific ROS. Cells were photographed using 100x magnification with a fluorescence microscope. The mean fluorescence intensity was quantified per image field using image J for all probes and the scale bar in representative images correspond to 50µm. **(F)** Left bar graph: RT-qPCR analysis of *NFE2L2*, *NQO1*, *SOD2*, and *GPX1* gene expression in shScrm and ShPDlim2 cells following culture for 48 hours. A representative western blot showing PDLIM2, catalase, HO-1, SOD2, and Tom20 protein expression in these cells. Right bar graph shows protein expression normalized against levels of Catalase, SOD2 or HO- in ShScrm cells, set to 1. **(G)** ShScrm and shPDLIM2 Caco-2 cells were cultured in the presence of 10µM MG132 for 4 hours and then stimulated with 5µg/mL fibronectin for 15 minutes prior to cell lysis and western blotting to quantify expression levels of NRF2, FAK, p-FAK (Y397), and PDLIM2. Bar graphs on right show fold change in NRF-2 and p-FAK expression, respectively. **(H)** Cells were incubated with CellROX orange for 30 minutes prior to stimulation with 5µg/mL fibronectin for 0, 15, 30, 60, or 120-minutes and photographed using 100x magnification using a fluorescence microscope. The bar graph shoes the mean ± SEM of CellROX staining from three independent experiments. All bar graphs depict the mean ± SEM of three independent experiments. P-values were calculated using the student’s t-test and where * = P < 0.05, ** = P < 0.01, and *** = P < 0.001.

During the selection and culture of Caco-2 cells with PDLIM2 stably suppressed (shPDLIM2) it was noted that the culture medium became highly acidified, particularly as the cells reached confluency. This suggested a metabolic switch following PDLIM2 suppression that may be related to cell adhesion, which we investigated further. Elevated glycolysis was indicated by higher protein levels of the Glut-1 glucose transporter (**Figure 6A**), and mRNA levels of *hexokinase 2 (HK2)* and *BACH1* (**Figure 6B**), both of which were significantly higher in shPDLIM2 cells than shScrm cells. Phosphorylation of AMP-activated protein kinase (AMPK) on Threonine 172 was also significantly higher in shPDLIM2 cells than in control shScrm cells cultured either in high or low glucose-containing medium, indicating metabolic stress (**Figure 6C**). This prompted us to investigate mitochondrial function and oxidative stress in shPDLIM2 cells and controls. First, we observed that mitochondrial membrane potential, assessed by TMRE staining, was significantly elevated in shPDLIM2 cells compared to shScrm cells (**Figure 6D**). Cellular ROS levels were quantified using H_2_DCFDA as a hydrogen peroxide probe, CellROX to detect superoxide, and MitoSOX to detect mitochondria ROS. These analyses (**Figure 6E**) demonstrated that shPDLIM2 cells exhibited higher levels of ROS detected by all three probes, with the high CellROX and MitoSOX signals indicating predominantly mitochondria-derived ROS.

The levels of H_2_DCFDA-detected ROS products suggested that impaired antioxidant buffering may accompany the observed elevated mitochondria-derived ROS. To test this, we investigated the expression of key genes in the antioxidant response pathways by qPCR. Levels of *NFE2L2* and *GPX1* were significantly lower in shPDLIM2 cells than shScrm controls, while *SOD2* trended lower and *NQO1 levels* trended higher in shPDLIM2 cells than controls (**Figure 6F**). Protein expression levels of catalase, HO-1, and SOD2, were also lower in shPDLIM2 cells than in controls (**Figure 6F**). However, despite the higher levels of ROS in shPDLIM2 cells NRF2 protein expression was not observed to be higher (**Figure 6G**). NRF2 protein accumulated in shPDLIM2 cells in the presence of the proteasomal inhibitor MG132, suggesting that PDLIM2 suppression decreases the stability of NRF2. Engagement with fibronectin for 15 minutes further increased NRF2 accumulation in the presence of MG132 in control cells, but decreased its accumulation in shPDLIM2 cells. Dephosphorylation of Focal Adhesion Kinase (FAK) was used as a control for integrin engagement (**Figure 6G**).

This result suggests that PDLIM2 regulates the stabilization and turnover of NRF2 in response to adhesion signals. In line with reduced NRF2 stability, cellular ROS levels were also observed to increase following fibronectin stimulation, which was more evident in shPDLIM2 at 15 minutes than in controls and was maintained up to 120 minutes (**Figure 6H**).

Taken together, these data indicate that PDLIM2 suppression results in impaired adhesion signalling, metabolic stress, sustained ROS production and impaired anti-oxidant buffering in response to adhesion signalling.

### PDLIM2 is suppressed in human ulcerative colitis along with alterations in cellular adhesion and inflammation

Our observations thus far support an essential function for PDLIM2 in maintaining intestinal epithelial tissue and cell homeostasis, so we were interested to determine whether this may extend to human colitis. To this end, we interrogated the publicly available dataset generated by Smillie et al., from single-cell RNA sequencing of 336,650 cells isolated from 68 colon biopsies from healthy individuals, or patients with uninflamed and inflamed ulcerative colitis. The single-cell profiles were clustered into 51 subsets by their known phenotypic markers, identifying 15 epithelial clusters, 13 stromal and glial clusters, and 23 immune cell clusters (21).

**Figure 7:**
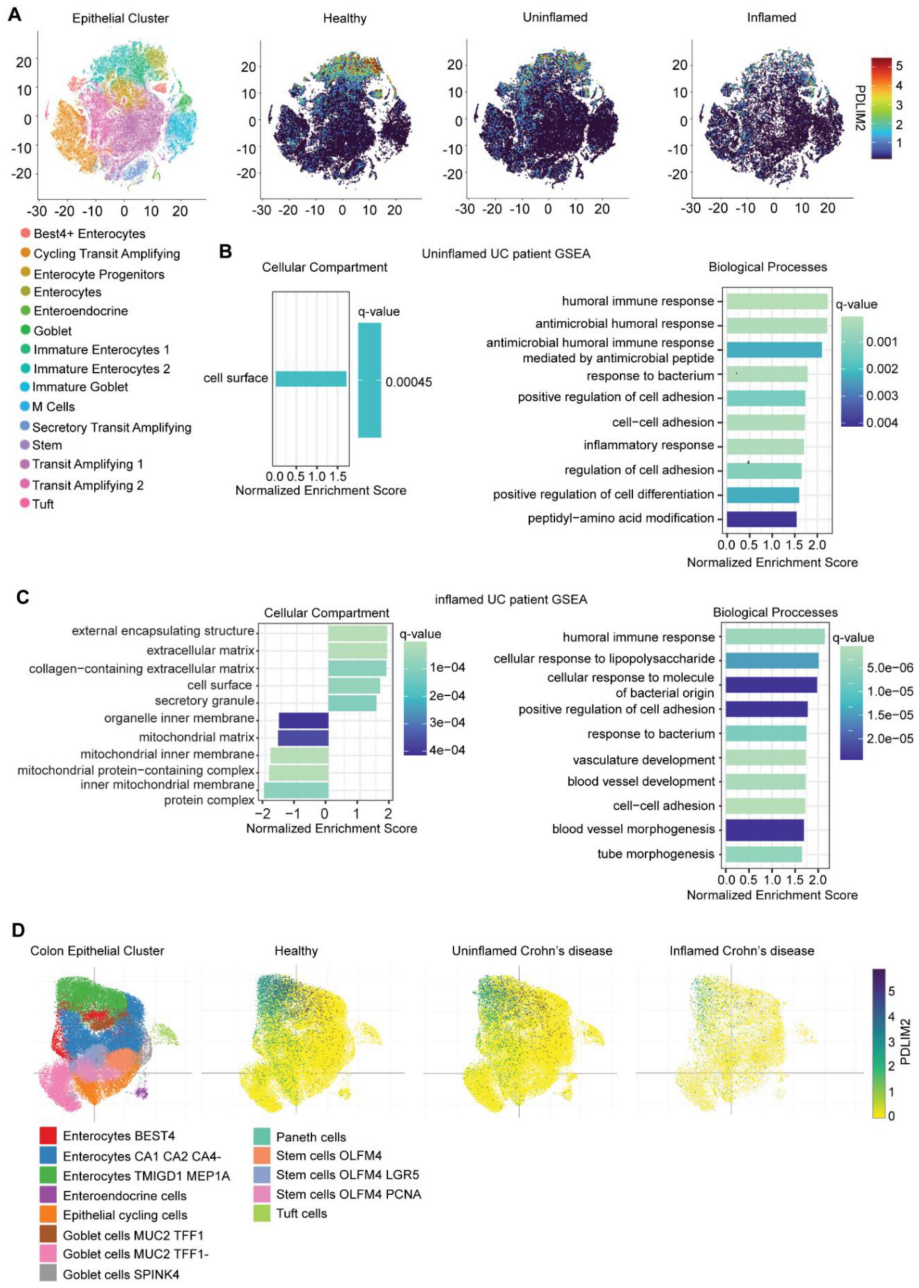
PDLIM2 expression declines in enterocyte population during IBD, corresponding with enrichment of markers for cell adhesion and impaired mitochondrial function. **(A)** t-SNE plot of epithelial populations were generated from the single-cell RNA-sequencing dataset derived from healthy and inflamed/uninflamed ulcerative colitis (UC) colonic biopsies. Each epithelial population is represented by color. t-SNE plots of healthy, uninflamed UC samples, and inflamed UC samples displaying the relative gene expression of *PDLIM2*. Relative gene expression is depicted by heatmap expression where the highest expression (5) is depicted in red while the lowest (0) corresponds to black. **(B)** Differential expression analysis was conducted on the enterocyte population comparing uninflamed UC patient samples to healthy volunteers for gene set enrichment analysis (GSEA). GSEA of cellular compartments and biological pathways enriched in uninflamed UC patient samples is represented by bar-plots highlighting the top 10 enriched pathways. **(C)** GSEA analysis was conducted on differentially expressed genes in inflamed patients compared to healthy volunteers and the top 10 enriched cellular compartments and biological pathways altered were represented as bar plots. **(D)** t-SNE plots of epithelial populations from colon samples of healthy and uninflamed/inflamed Crohn’s disease patients were acquired directly from the Broad institute’s single cell portal. Plots represent the relative gene expression of *PDLIM2* as depicted by heatmap expression where 0 is depicted as yellow and 5 corresponds to navy.

We analysed *PDLIM2* expression in t-SNE plots of epithelial cells (**Figure 7A**). Within the epithelial populations, *PDLIM2* was highly enriched in mature enterocytes in healthy volunteers consistent with the profiles observed in mouse colon tissue (**Figure 2A**). However, *PDLIM2* expression was lower in both uninflamed and inflamed enterocyte populations of patients with ulcerative colitis than in tissue from healthy individuals (**Figure 7A**).

Since PDLIM2 was highly expressed within the enterocyte population of healthy individuals, while its expression was reduced in uninflamed and inflamed tissues of patients with ulcerative colitis, we used GSEA to investigate which cellular compartments and biological pathways were altered. In uninflamed tissues, only genes associated with the cell surface were considered to be significantly altered, with relevant biological pathway enrichment corresponding to immune responses and cellular adhesion (**Figure 7B**). In inflamed tissues, genes associated with the extracellular matrix and cell surface were observed to be positively enriched, while those attributed to the mitochondrial matrix and mitochondrial protein complex were negatively enriched (**Figure 7C**). Furthermore, as previously observed in the uninflamed tissue, biological pathways associated with immune responses and cellular adhesion were positively enriched (**Figure 7C**).

Interestingly, in a similar study where 720,633 single-cell transcriptomes were generated from colon and terminal ileum tissues of healthy and Crohn’s disease patients and devised into epithelial (97,788 and 154,136), stromal (39,433 and 75,695), and immune (152,509 and 201,072) cell clusters, *PDLIM2* expression was also shown to be suppressed specifically in uninflamed and inflamed colon tissues of patients with Crohn’s disease (**Figure 7D**).

Taken together, the data demonstrate that PDLIM2 is predominately expressed by the enterocyte population in comparison to other cell types in the human intestine. In the colon, the suppression observed in murine models is reflective of data from patients with both inactive and active colitis and Crohn’s disease colitis. The observed altered adhesion signalling and mitochondrial transcriptome is also in agreement with our observations following PDLIM2 suppression in Caco-2 cells (**Figures 5**, **6**).

## Discussion

**Figure 8:**
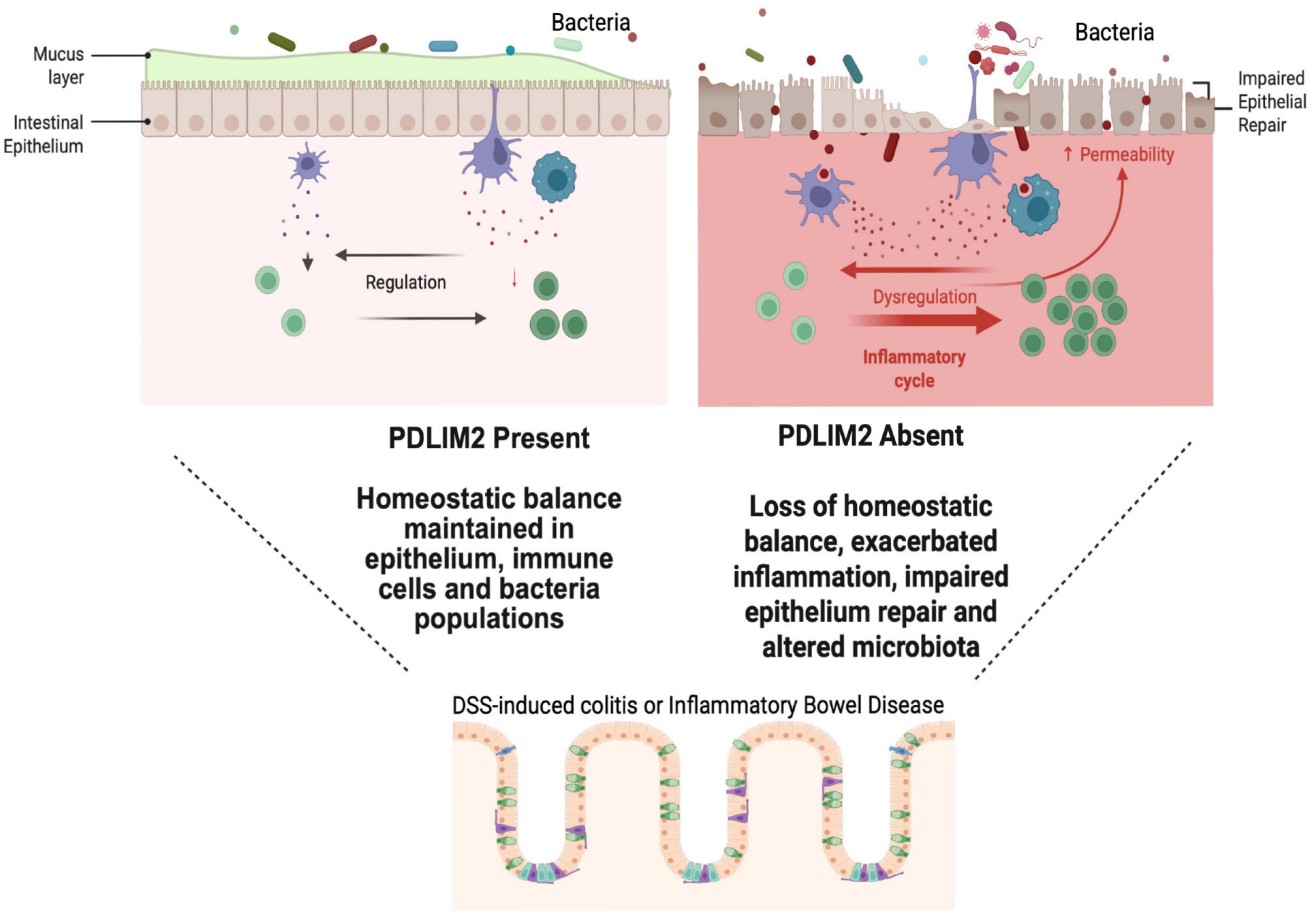
Summary model incorporating the findings from murine colitis model, human RNA seq datasets and analysis of cell cultures. PDLIM2 -/- mice have altered basal microbiota compared to their +/+ counterparts and exhibit greatly exacerbated inflammation and lack of epithelium recovery in response to DSS. PDLIM2 expression in mouse and human enterocytes is lost as colitis progresses, and cells deficient in PDLIM2 have elevated oxidative stress as well as impaired epithelial cell polarity, migratory and adhesion capacity. Image generated in Biorender.

As a cytoskeleton and nuclear protein PDLIM2 regulates cell adhesion signalling and the stability and function of transcription factors that control cell fate and differentiation in hematopoietic and epithelial cells (4, 5, 7, 14, 15, 25, 26). Our findings summarized in a model (**Figure 8**) demonstrate that the presence of PDLIM2 is required to maintain intestinal integrity in the presence of inflammation associated with colitis. The absence of PDLIM2 favours the expansion of pro-inflammatory immune populations, which is in line with the known PDLIM2 function of limiting pro-inflammatory STAT and NF-κB signalling pathway responses. Importantly, the absence of PDLIM2 alone is not sufficient to promote inflammation, because PDLIM2 -/- mice did not demonstrate any overt inflammatory phenotype compared to their +/+ counterparts. However, in response to DSS-induced intestinal injury, PDLIM2 -/- mice exhibited persistently high infiltration of immune cell populations and pro-inflammatory cytokine expression, accompanied by reduced infiltration of CD206+ wound-healing macrophages. Thus, the feedback limitation of pro-inflammatory signalling is severely impaired in PDLIM2 -/- mice.

The absence of PDLIM2 resulted in apparently weakened epithelial integrity and disrupted epithelial architecture in PDLIM2 -/- mice exposed to DSS. Interestingly, in PDLIM2 +/+ mice with repeated exposure to DSS, PDLIM2 protein levels became suppressed not only during active inflammation but remained suppressed following apparent epithelial regeneration. Importantly, a similar repression of PDLIM2 was observed in human enterocytes in single cell RNA seq datasets from colon samples from patients with ulcerative colitis or Crohn’s disease at both inactive and active stages of the diseases. Together, this suggests that PDLIM2 loss may sensitize the colon epithelium to injury. Given the prevalence of PDLIM2 expression within the enterocyte population, we propose that PDLIM2 is required both for maintaining gut epithelial integrity and for limiting pro-inflammatory immune responses. The sustained repression of PDLIM2 may also render the colon susceptible to oncogenic transformation, as was previously demonstrated in cell and models of PDLIM2 deficiency (8, 27). However, although our intitial experimental setup included conditions for progression to CAC in response to AOM+DSS, we were unable to test this concept directly because the study had to be halted due to the severe inflammation and clinical effects observed in PDLIM2 -/- mice following two cycles of DSS. Thus, there was not sufficient time for tumors to form, as was confirmed by pathology analysis.

Although PDLIM2 -/- mice do not normally present an overt inflammatory or other phenotype, it was interesting to find altered microbiota profiles in these mice. Lack of PDLIM2 appears sufficient to alter the diversity, composition and predicted functionality of the bacterial community present in the gut. These alterations were further noticeable in DSS-treated PDLIM2 -/- animals. In particular, PDLIM2 suppression and DSS treatment led to an increase in alpha diversity with a reduction in *Lactobacillus* (the dominant genus in healthy mice (28)), suggesting a disruption of the ecosystem with the emergence of typical subdominant taxa, some of which could be detrimental to gut health. For example, *Erysipelotrichaceae*, *Peptostreptococcaceae* and *Clostridia_UCG-014*, which characterized PDLIM2 -/- DSS mice, have previously been associated with gut dysbiosis and inflammation (29–31). Among them, *Romboutsia*, a genus of the *Peptostreptococcaceae* family that specifically discriminated PDLIM2 -/- DSS mice, has recently been suggested to play a pro-inflammatory role in DSS-induced colitis and ulcerative colitis (32, 33). Furthermore, the absence of PDLIM2 alone resulted in other potentially dysbiotic changes, including an increase in *Lachnospiraceae;*UCG 006, which has been associated with adverse health outcomes in mouse models (34), and *[Eubacterium]_xylanophilum_*group, a pathogenic gut bacterium positively associated with colitis in mice (34).

Functionally, PDLIM2 suppression and DSS treatment resulted in the potential loss of a number of microbial pathways related to basic cellular functions, offset by the gain of pathways related to the immune system, signal transduction, cell motility, antimicrobial resistance and pathogenic processes, which aligns with the observed sensitivity of these mice to epithelial damage and inflammation. Notably, the fatty acid β-oxidation I pathway, overrepresented in PDLIM2 -/- DSS mice, has previously been found to be enriched in the gut microbiota of patients with ulcerative colitis and linked to enhanced inflammatory responses (35). However, the present study cannot establish whether the microbiota is a key pathogenic driver of the extreme colitis in PDLIM2 -/- mice. Future studies, including fecal microbiota transplantation, may shed light on this issue and also explore the potential for targeting the microbiota therapeutically.

Although it is not possible to fully integrate the findings from the mouse model with the cell model and human colitis datasets, our observations collectively support an epitheilial-intrinsic function for PDLIM2 along with immune cell and microbiota alterations. PDLIM2 has a known function in maintaining epithelial cell polarity necessary for cell-cell interactions and directional motility (5). In Caco-2 cells when PDLIM2 was suppressed, impaired adhesion signalling was accompanied by metabolic stress and elevated mitochondria-derived ROS. A direct relationship between adhesion signalling and the regulation of metabolic signalling pathways has previously been described (36–38). Integrin activation and matrix stiffening support a shift towards glycolysis associated with enhanced stability of glycolytic enzymes and glucose importers (39), while levels of mitochondrial respiration complex components can be suppressed in the presence of elevated membrane potential and ROS production, thereby impairing mitochondrial function (39). Metabolic and oxidative regulation of HIF-1α, AMPK, and mTOR are also implicated in cell adhesion by controlling both the expression and activity of integrins (40). Future studies that would further interrogate these mechanisms could include the study of intestinal organoids derived from PDLIM2 -/- and +/+ mice in the presence or absence of inflmmmatory mediators. In this regard our preliminary unpublished studies with organoids support epitheium-intrinsic differences in PDLIM2 +/+ and PDLIM2 -/- tissues, with lower levels of epithelial and stem cells markers present in PDLIM2 -/- organoids.

This study did not directly address targeting approaches for mucosal healing and restoring homeostasis in IBD. However, the findings support further investigation of the relevance of restoring PDLIM2 expression and directly microbiome targeting as a therapy. Many interesting studies have identified mediators of inflammation, tissue damage and cell death in colitis. For example, TMEM219-mediated intestinal cell death death in Crohn’s disease (41), SOX6, F3 (CD142), and WNT genes derived from a fibroblast niche that supports intestinal stem cells function and epithelial regeneration (42). Macrophage-derived TIM-3 has also been implicated in protecting gut from inflammation induced damage during colitis (43). It is conceivable that PDLIM2 has a role in feedback regulation and unrestrained activation of some or all of these signalling mediators in colitis. The concept of restoring PDLIM2 expression has already been touched on. In a murine model of colitis, the micro-RNA-214 (miR-214) can suppress PDLIM2 expression, and it has been demonstrated that an miR-214 inhibitor reduces inflammation and increases PDLIM2 (44). In a cancer model, Sun et al. introduced PDLIM2 expression plasmids encapsulated within nanoparticles (nanoPDLIM2) to the mouse lung cancer model (45). NanoPDLIM2 did not have pathological effects on major organs, but had anti-tumor activity, and significantly improved the efficacy of anti-PD-1 immunotherapy and chemotherapeutic drugs (46).

In summary, our findings indicate that PDLIM2 loss exacerbates the progression of colitis in impairment of integral pathways involved in epithelial cell adhesion, polarization, and metabolic function. Although our study fell short of determining whether these effects of PDLIM2 suppression facilitate progression to cancer, it is clear that the absence of PDLIM2 impairs gut epithelium recovery and contributes to an environment that favours injury. Therefore, the colon may be an important site for the tumor suppressor functions of PDLIM2.

## Materials and methods

### Mice

PDLIM2 -/- were derived as previously described through successive backcrossing of BALBc mice with C57BL/6J mice (3, 14). All mice were bred as heterozygotes (PDLIM2 -/+) in individually ventilated cages (IVCs, OptiMICE), in a controlled environment (20–22 °C, 12 hours light:dark cycle) and given food and water *ad libitum* at the Bioscience Service Unit (BSU)-Annex at University College Cork. Mice (PDLIM2 +/+ and PDLIM2 -/-) were housed under specific pathogen-free standard conditions and maintained in accordance with the protocols and procedures approved by University College Cork Animal Experimentation Ethics Committee (AEEC, application #2017-022) and under the authorization from the Health Regulatory Authority of Ireland (HPRA authorization number: AE1930/P077).

### DSS-induced acute colitis and DSS+AOM-induced colitis-associated colorectal cancer

Both male and female mice were used for the studies with DSS (acute/chronic) and CAC. To induce acute colitis, mice (aged 9–15 weeks) were administered drinking water containing dissolved 2.5% w/v DSS (TdB consultancy, Cat # DB001, 40kDa DSS) for 5 days followed by normal drinking water for 3 days (47). The DSS solution was freshly prepared every second day.

For induction of chronic colitis and CAC, a combination of azoxymethane (AOM) (Merck, Cat # A5486) followed by cycles of DSS was used (48–50). Mice (ages 10–12 weeks) were injected intraperitoneally with a single dose of 10 mg/kg body weight of AOM (day -7). After seven days (day 0), mice received drinking water containing 2% w/v DSS for 5 days followed by normal drinking water for 14 days. This cycle was repeated once, and although a third cycle was planned, this was not carried out due to the adverse symptoms in PDLIM2 -/- mice, and the experiment was terminated on day 38, when all mice were sacrificed by cervical dislocation followed by verification of death by palpation. Tissues (colon, spleen) and fecal samples were immediately harvested and processed as outlined below.

Mice were individually monitored daily for clinical symptoms and scores recorded for the symptoms according to the following scales: body weight loss (0–3), stool consistency (0-3), posture (0-2), fur texture (0-2), and rectal bleeding (0-2) (51), with some modifications. In brief, weight loss score was defined as follows: 0 = up to 5% loss, 1 = 6% to 15% loss, 2 = 15% to 25% loss, and 3 = more than 25% loss. For stool consistency: a score of 0 = normal pellet, 1 = changed form of pellets, 2 = loose stool, and 3 = diarrhea. For fur and posture scoring: 0 = smooth fur and no hunch, 1 = mildly scruffy and hunched, and 2 = very scruffy and hunched. For rectal bleeding: a score of 0 = no blood, 1 = blood in feces, and 2 = blood alone. A daily disease activity index (DDAI) was generated for each study cohort by obtaining the mean and standard deviation from the sum of scores for each mouse in the cohort.

### Mouse colon tissue preparation and histology

Intact colons were removed from mice, washed with sterile PBS, dimensions recorded and cut longitudinally to divide the proximal and distal colon as described (51). Briefly, three cm of the distal colon tissue was divided into three sections; 0.5 cm of the most distal end was frozen for RNA extraction, the remaining 2.5 cm was sectioned longitudinally and rolled (“swiss rolled”) before freezing in optimal cutting temperature (OCT) cryostat embedding matrix (Cat # 361603E, VWR) using liquid nitrogen. For histology and immunofluorescence, frozen sections (8 µm) were cut using a cryostat (Leica) and collected on Superfrost glass slides (Fisher Scientific). Sections were air-dried and fixed in Tris Buffered Saline (TBS) containing 4% paraformaldehyde (PFA) and then stained with haematoxylin and eosin (H+E). All stained sections were anonymized, and the scores for histological scoring determined as previously described (52). Briefly, no epithelial degeneration and few inflammatory cells was defined as 0, few signs of epithelial degeneration and mild inflammation as 1, few signs of epithelial ulceration and mild inflammation as 2, few signs of epithelial ulceration and moderate inflammation as 3, ulceration in more than 25% of the tissue section and moderate to severe inflammation as 4, ulceration of more than 50% of the tissue sections and moderate to severe inflammation as 5, and ulceration of more than 75% of the tissue sections and severe inflammation as 6. All sections from chronic colitis and CAC groups were independently clinically assessed to determine whether tumors were present.

### Immunofluorescence of frozen tissue sections

The following primary antibodies were used: rat anti-F4/80 (#123102, 1:100), rat anti-CD206 (#141702, 1:100), and rat anti-CD8 (#100702, 1:100) all from Biolegend. The rabbit anti-F4/80 (#30325, 1:400), rabbit anti-Ki67 (#12202, 1:500), and rabbit anti-E-Cadherin (#3195, 1:400) antibodies were acquired from Cell Signaling, while rabbit anti-CD3 (#16669, 1:100) and mouse anti-PDLIM2 antibodies (#68220, 1:200) were acquired from Abcam. Finally, rabbit anti-ZO-1 antibody (40-2200, 1:100) was acquired from Fisher Scientific Ireland Ltd and goat anti-PDLIM2 antibody (#EB11878, 1:500) from Generon. Secondary antibody Alexa Fluor 488 donkey anti-rat antibody (#150153) was acquired from Abcam while Alexa Fluor 488 donkey anti-rabbit (711-545-152), Cy3-conjugated donkey anti-rabbit (711-165-152), Alexa Fluor 488 donkey anti-goat (705-545-147), and Cy3-conjugated donkey anti-mouse antibodies (715-165-150) were all acquired from Jackson ImmunoResearch Europe.

Frozen tissue sections were air-dried, fixed in 4% PFA/ice-cold methanol followed by permeabilization and blocking in Tris buffered saline (TBS) containing 10% donkey serum (DS), 1% bovine serum albumin (BSA) and 0.1% Triton X-100 for 45 minutes. Sections were then incubated with primary antibodies at the indicated dilutions in TBS containing 5% DS and 1% BSA for 18 hours at 4 °C. Following three washes with TBS, slides were incubated with Alexa 488- and/or Cy3-conjugated secondary antibodies and Hoechst 3342 nuclear stain for 90 minutes. For PDLIM2 staining, sections were blocked in 5% DS and 0.01% saponin dissolved in TBS for 60 minutes. Sections were incubated in primary antibody overnight in TBS containing 1% DS and 0.01% saponin. Sections were washed with TBS/1% DS/0.01% saponin three times and incubated in secondary antibody and Hoechst dye for 30 minutes. Sections were washed three times and mounted with vinol mounting medium (15% w/v Celvol #205s with 33% v/v glycerol and 0.1% azide in PBS) before applying coverslips. Images were acquired using a Licor DM LB2 microscope with a Nikon Digital Sight DS-U2 camera. A minimum of 7 images per field were analyzed for each tissue section, and images were obtained at 40x magnification. Quantification of immunofluorescence was carried out using the Image J imaging software (NIH).

### RNA extraction and RT-qPCR

MagNA Lyser green beads (Roche Diagnostic) were used to homogenize 30 mg of colon tissue for RNA extraction using GeneJet RNA extraction kits (Fisher Scientific), as previously described (53). RNA isolation from cell line culture was carried out following instructions in the GeneJet RNA extraction kit. cDNA was prepared using QuantiTect reverse transcription kit (QIAGEN), and quantitative Real-Time PCR (qRT-PCR) was carried out using FastStart Essential DNA Green Master mix (Roche Diagnostics) and a Roche Lightcycler 96 (Roche Diagnostic). To quantify gene expression, the cycle of threshold value (CT) of the housekeeping gene was subtracted from the gene of interest to acquire ΔCT. The raw value of the gene of interest (2^ΔCT^) was acquired using the formula =SUM(POWER(2, -ΔCT)) and then normalized to the control untreated sample to find the fold-change difference in mRNA expression. All primers were obtained by Integrated DNA Technologies (IDT) and are listed in **Supplementary Table 1**.

### Cell culture

Caco-2 cells were obtained from ATCC and cultured in Dulbecco’s Modified Eagles media (DMEM) containing 10% fetal bovine serum and 1% L-glutamine, and 1% penicillin/streptomycin. Cells were cultured at 37 °C and in atmosphere of 5% CO_2_. To suppress PDLIM2 in the Caco-2 cell-line, cells were transfected with the pSuper.neo RNAi vector containing a shScrm (TGACATGATAATACTCTCT) insert or shPDLIM2 (ACATAATCGTGGCCATCAA) insert followed by selection using Geneticin. Pools of cells with stable PDLIM2 suppression were maintained on tissue culture plates coated with 50µg/mL collagen-I (Gibco #10677174) to support the adhesion of cells with PDLIM2 suppressed.

To culture cells in low glucose DMEM from Merck (#D6046), Caco-2 cells were seeded at 6x10^5^ per 6-well plate overnight in high-glucose medium (4.5g/L). Cells were then washed 3x in PBS to remove residual high glucose before being cultured for an additional 48hrs in low glucose medium (1g/L).

### Mitochondrial membrane potential

Caco-2 cells were seeded at 2 × 10^5^ per well in 6-well plates and allowed to reach 50% confluency. Cells were incubated in 500 nM TMRE dye (Fisher Scientific #11650796) for 20 minutes at 37 °C in the dark. Cells were then washed three times in PBS and detached using accutase. Cells were centrifuged at 1000 rpm for 5 minutes to remove accutase and resuspended in phenol-free DMEM before flow cytometry with a BD Accuri C6 Plus instrument. Unstained cells served as a negative control for fluorescence, while cells incubated in 20 µM of CCCP (Merck #C2759) for 20 minutes prior to flow cytometry analysis served as controls for TMRE staining. Data were analyzed using FlowJo V10 analysis software to calculate the geometric mean.

### Cellular reactive oxygen species analysis

Caco-2 cells were seeded overnight at 1 × 10^5^ per well in CELLview 4 compartment glass bottom plates from VWR (#391-0253). Adherent cells were washed once in PBS and incubated with 5 µM of CellROX orange from Fisher Scientific (#C10443) for 30 minutes in the dark to detect basal cellular ROS. To measure cellular ROS upon integrin engagement, cells were initially incubated with CellROX orange for 30 minutes in the dark before stimulation with 5 µg/mL of fibronectin. Images were acquired at 15-, 30-, 60-, and 120-minutes post-stimulation using 100x magnification with oil immersion on a Nikon Eclipse TE300 inverted microscope and Nikon Intensilight C-HGFI light source with a Hamamatsu C11440 Digital Camera and Nikon Image Suite Elements AR software. To assess mitochondria-specific ROS, Caco-2 cells were stained with 500 nM MitoSOX red from Fisher Scientific (#M36008) for 10 minutes in the dark. Cells were washed three times with PBS and photographed at 100x magnification using oil immersion. To quantify hydrogen peroxide species, Caco-2 cells were incubated in complete medium containing 10 µM of H_2_DCFDA for 30 minutes in the dark, washed three times with PBS, and photographed at 100x magnification with oil immersion. For all images, the fluorescence intensity was quantified using ImageJ analysis software.

### SDS page electrophoresis and western blotting

Caco-2 cells were lysed using RIPA lysis buffer (50 mM Tris pH 8.0, 150 mM NaCl, 1% NP40, 0.5% sodium deoxycholate, 0.1% SDS) with phosphatase (sodium orthovanadate, sodium pyrophosphate, β-glycerophosphate) and Pierce Halt protease inhibitors (AEBSF, aprotinin, bestatin, E64, leupeptin, pepstatin A). Proteins were resolved by SDS-PAGE gel electrophoresis using 4-20% gradient gels and transferred to nitrocellulose membranes for 70 minutes at constant 250 mAMP. Blots were probed using the following antibodies:

Rabbit anti-NRF2 (#12721), Rabbit anti-FAK (Y397) (#8556), rabbit anti-HO-1 (5061), rabbit anti-catalase (#14097), rabbit anti-AMPK (#2532), rabbit anti-p-AMPK (T172) (#2535), rabbit anti-GAPDH (2188), mouse anti-ubiquitin (#3936), and rabbit anti-β-catenin (#8480) were acquired from Cell Signaling. From Santa Cruz Biotechnology rabbit anti-Tom20 (sc-17764), and mouse anti-α-Tubulin (#5286) were acquired. Mouse anti-β-actin (A5441) was acquired from Merck. The mouse anti-E-Cadherin (#610181) was acquired from BD and mouse anti-PDLIM2 (#68220), rabbit anti-SOD2 (#68155) and rabbit anti-Glut-1 (#652) were acquired from Abcam. Rat anti-FAK (#694001) was acquired from BioLegend and rabbit anti-β1-integrin (#1952) was acquired from Millipore.

All primary antibodies were used at 1:1000 dilution except for β-actin (1:10,000). LI-COR Biosciences secondary antibodies, IRDye680 goat anti-mouse (926-68070), IRDye800 goat anti-rabbit (925-32211), or IRDye800 donkey anti-goat (925-32214) were used at 1:10,000 dilution. Western blots were scanned using the Odyssey scanner (LI-COR Biosciences) and fluorescence signals in each channel (680 red, 800 green) quantified using Image Studio Lite Version 5.2 analysis software.

To assess protein stability of β1-integrin, Caco-2 cells were cultured in the presence of 10 µM of MG132 (Merck #474790) or 10 nM of Bafilomycin A1 (MedChemExpress #HY-100558) for 14 hours prior to lysis.

### Cell motility assays

Caco-2 cells (5 × 10^4^) were seeded into Ibidi insert chambers (Thistle Scientific #80209) in a 24-well plate and allowed to adhere overnight. Inserts was then removed, and cells were gently washed in PBS. Fresh medium were added, and images were acquired using the Eclipse TE300 inverted microscope at 10x magnification. Multiple images were acquired at times 0- and 20-hours following insert removal. Wound filling analysis were conducted using the Image J processing software.

### Cell adhesion assays

Wells of a 24 well plate were pre-coated for 60 minutes at 37 °C with collagen-1 (50 µg/mL dissolved in 20 mM acetic acid) or fibronectin (5 µg/mL in PBS). Plates were washed 3 times in PBS and blocked for an additional 60 minutes with 2.5% BSA. Caco-2 cells were seeded at a density of 2 × 10^5^ per well for 30 minutes, 60 minutes, or 120 minutes for fibronectin-coated plates and 15 minutes, 30 minutes, or 60 minutes on collagen-1-coated plates. Non-adhered cells were removed by washing with PBS and adhered cells were fixed for 5 minutes in 100% ice-cold methanol. The cells were stained for 15 minutes in 0.05% crystal violet dissolved in 20% ethanol, washed in dH_2_0 to remove excess crystal violet, and allowed to dry. Images were acquired using the Nikon Eclipse TE300 inverted microscope and Nikon Intensilight C-HGFI light source with a Hamamatsu C11440 Digital Camera with Nikon Image Suite Elements AR software at 20x magnification. Crystal violet staining was quantified using Image J processing software.

### 16S rRNA amplicon sequencing for fecal microbiota profiling

Microbial DNA was extracted from stool samples using the QIAamp DNA Stool Mini Kit (QIAGEN) according to the manufacturer’s instructions. Library preparation was performed as described in the Illumina protocol “16S Metagenomic Sequencing Library Preparation” (Illumina). Briefly, the V3–V4 hypervariable region of the 16S rRNA gene was amplified using the 341F and 785R primers containing Illumina adapter overhang sequences (54).

Amplification was performed using KAPA Hi Fi HotStart ReadyMix (Roche) with the following thermal cycle: 3 minutes at 95 °C, 25 cycles of 30 seconds at 95 °C, 30 seconds at 55 °C, and 30 seconds at 72 °C, and a final 5-minute step at 72 °C. Amplicons were purified using a magnetic bead-based clean-up system (Agencourt AMPure XP; Beckman Coulter). Indexed libraries were prepared by limited-cycle PCR using Nextera technology, followed by a second clean-up step as described above. The final library, prepared by pooling samples to an equimolar concentration of 4 nM, was denatured and diluted to 5 pM with a 20% PhiX control. Sequencing was performed on an Illumina MiSeq platform using a 2 × 250 bp paired-end protocol, as per manufacturer’s guidelines. Sequencing reads were deposited in the National Center for Biotechnology Information Sequence Read Archive (NCBI SRA; BioProject: PRJNA1192040).

Raw reads were processed using a pipeline combining PANDAseq (55) and QIIME 2 (56). After filtering for length (minimum/maximum = 350/550 bp) and quality (default parameters), reads were binned into amplicon sequence variants (ASVs) using DADA2 (57). Taxonomy was assigned using the VSEARCH algorithm (58), with the Silva database as the reference (March 2023 release). Alpha diversity was assessed using the Shannon index and the number of observed ASVs. Beta diversity was estimated by computing weighted and unweighted UniFrac distances, which were used to construct principal coordinates analysis (PCoA) graphs. PICRUSt2 (59) was used to predict the functional content of inferred synthetic metagenomes.

### Single-cell sequencing - t-SNE and differential expression analysis of ulcerative colitis patients

Single-cell data in the form of pre-normalized cell-gene matrices from Smillie et al. were acquired from the Broad Institute’s single cell portal) and github repository (https://github.com/cssmillie/ulcerative_colitis). Generation of t-distributed stochastic neighbour embedding (t-SNE) visualization for the Smillie et al. (21), dataset was achieved as previously described (60). Briefly, epithelial matrices and metadata files were used to create a Seurat (61) object in R and the normalized counts of PDLIM2 transcript were assessed across cell populations. Figures were plotted using ggplot2 (62) and colour scales used for gene expression were produced using the viridis package (63).

Differential expression analysis was performed as outlined in Smilie et al. (21). In brief, a hurdle model against the expression of a gene in both the linear regression for the continuous process (referring to the level of expression of a gene) and the logistic regression for the zero process (referring to whether a gene is expressed or not) was performed to compare the three disease states (healthy, uninflamed, and inflamed) using MAST (64). Gene set enrichment analysis (GSEA) of differentially expressed genes in the enterocyte population of both uninflamed and inflamed ulcerative colitis patients compared to healthy controls was subsequently conducted using the Clusterprofiler package fitted in accordance to their distinct coefficient (65). GSEA was conducted on differentially expressed genes with all genes being ranked based on highest to lowest expression and any genes outside the p-value cut-off of 0.05 being omitted prior to enrichment analysis. Plots of GSEA were generated using ggplot2 and enrichplot packages (66).

t-SNE plots of *PDLIM2* expression were directly acquired from the Broad Institute’s single cell portal (https://singlecell.broadinstitute.org/single_cell/study/SCP1884/human-cd-atlas-study-between-colon-and-terminal-ileum) for epithelial populations of colon samples of healthy volunteers and the uninflamed and inflamed tissues of Crohn’s disease patients characterized by single-cell RNA sequencing data (22).

### Statistical analysis

For all data from the mouse cohorts, significance was assessed using one-way analysis of variance between cohorts with a Bonferroni correction applied. Unpaired student t-tests were used for calculating significance from western blot, qRT-PCR, and cell-based assays. P-values are indicated in the figure legend with p<0.05 considered statistically significant. All graphs were prepared, and statistical analysis conducted using the GraphPad Prism 5.03 software.

For the gut microbiota, all statistical analyses were performed using R software (version 4.2.2, https://www.r-project.org/). PCoA plots were generated using the vegan package (version 2.6-6.1, https://cran.r-project.org/web/packages/vegan/index.html), and data separation was tested using PERMANOVA (Adonis function in vegan). Kruskal-Wallis tests followed by *post-hoc* Wilcoxon tests were used to assess differences in alpha diversity, relative abundances at the phylum, family and genus level, and the abundance of predicted KOs (KEGG orthology) and inferred MetaCyc pathways between groups. Boxplots were generated using the packages ggplot2 (62) and ggsignif (67). Genus-level and predicted KOs heatmaps were generated using made4 (version 3.19, https://bioconductor.org/packages/release/bioc/html/made4.html) and the heatmap.2 function from the gplots package (version 3.1.3.1, https://cran.r-project.org/web/packages/gplots/index.html). For hierarchical clustering of selected genera and KOs, Ward’s linkage method was applied to the distance matrix derived from Spearman correlation coefficients. Genera discriminating between groups were identified using the linear discriminant analysis (LDA) effect size (LEfSe) algorithm (68). Only taxa with LDA score >2 at p-value <0.05 were considered. P-values were corrected for multiple comparisons using the Benjamini–Hochberg method where appropriate. A false discovery rate (FDR) ≤0.05 was considered statistically significant, while a p-value between 0.05 and 0.1 was considered a trend.

## Data Availability

The original contributions presented in the study are included in the article/**Supplementary Material**. Further inquiries can be directed to the corresponding author.
