# Supplementary material for: Loss of the PDLIM2 protein during chronic colitis promotes inflammation, impaired epithelium recovery, alterations to the microbiome and oxidative stress
Source: Front Endocrinol (Lausanne). 2026 Feb 10;16:1720162. doi: 10.3389/fendo.2025.1720162 (PMC12929155; doi:10.3389/fendo.2025.1720162)
Supplement: Supplementary file 1 [file DataSheet1.pdf]

Supplementary Material

Supplementary Figure 1

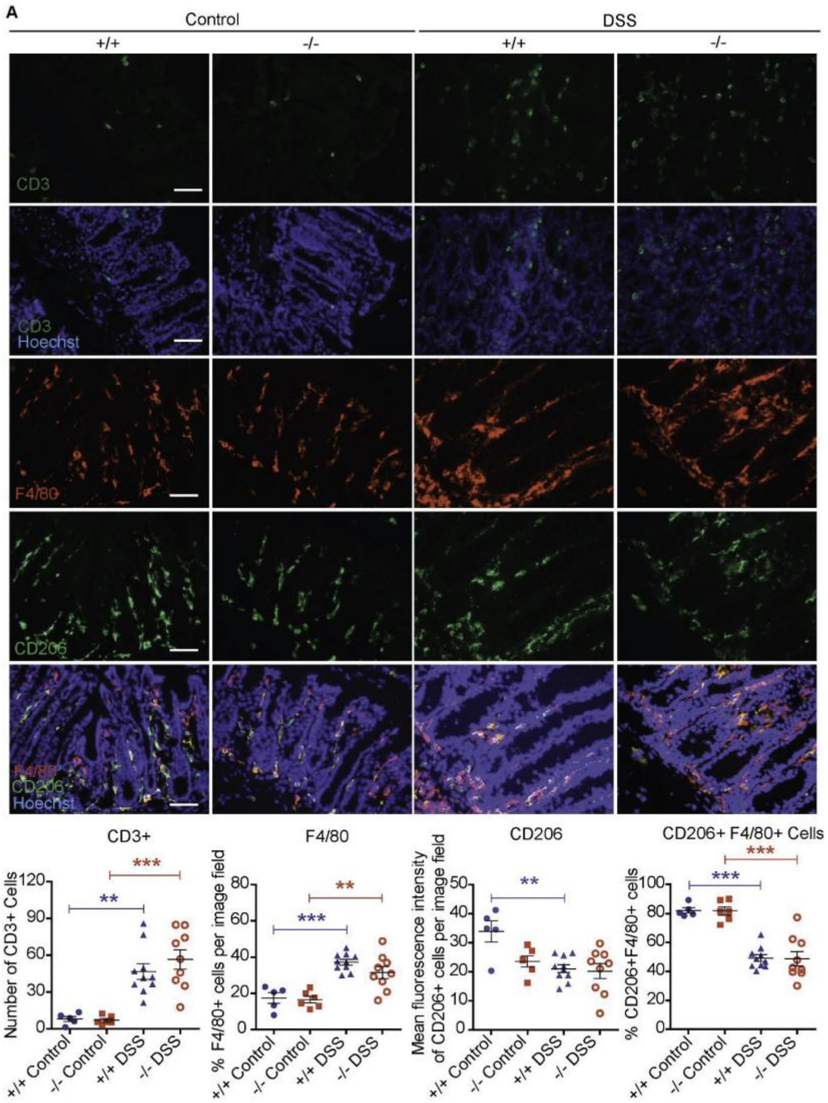

Similar levels of immune cell infiltration in DSS treated PDLIM2 +/+ and PDLIM2 -/- mice in acute colitis model. Histological tissue sections were stained with an anti-CD3 antibody (green) and Hoechst nuclear counterstain. Representative images were acquired at 40x magnification with scale bars representing 50µm. Quantification analysis was conducted by counting the number of CD3-positive (+) cells per image field (A). Tissue sections were co-stained with the pan macrophage marker F4/80 (red) and the M2 macrophage marker, CD206 (green) followed by Hoechst for nuclear counterstain. Images were acquired at 40x magnification with scaling representing 50µm. Quantification of the percentage of positive staining for F4/80, the level of CD206 expressed based on fluorescence intensity of the percentage of CD206 + cells present, and the percentage of CD206 + cells as a fraction of the total level of F4/80+ macrophages was conducted per image field (A). Statistical analysis between groups was calculated by one-way ANOVA with Bonferroni post-hoc

analysis. The asterisk (*) represent significant values where * $P < 0.05$, ** $P < 0.01$, and *** $P < 0.001$.

(B)

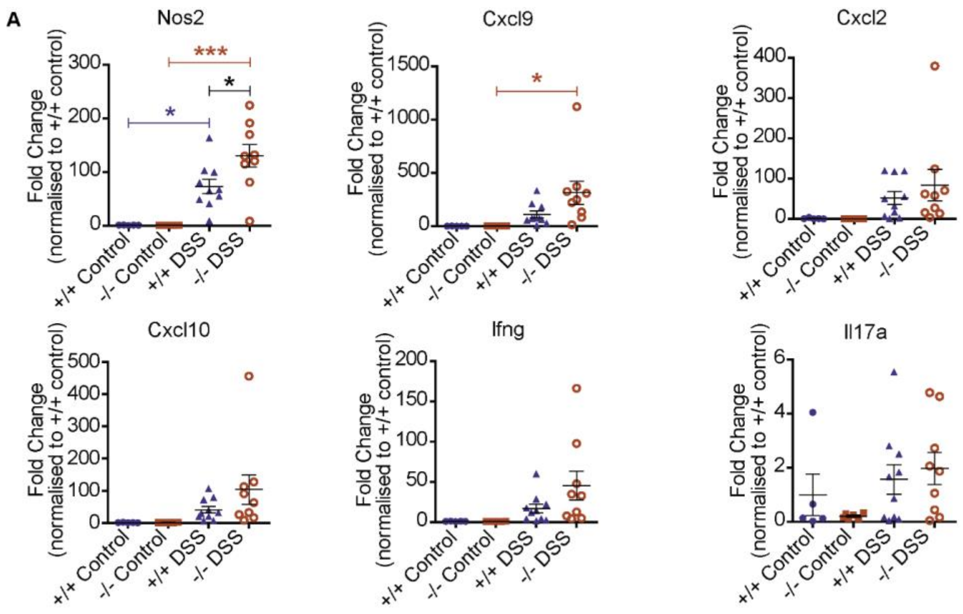

(B) PDLIM2-/- mice trend towards increased expression in pro-inflammatory genes.

Gene expression was determined from RNA derived from distal colon tissue by RT-qPCR analysis. Genes analysed were *Nos2*, *Ifng*, *Cxcl2*, *Cxcl9*, and *Cxcl10* Scatter plots were generated with mean ± SEM per group and significance calculated using one-way ANOVA and Bonferroni post-hoc (A). * $P < 0.05$, ** $P < 0.01$, and *** $P < 0.001$.

Supplementary Figure 2

Disease symptoms and tissue analysis of AOM+DSS-treated PDLIM2 +/+ and -/- mice

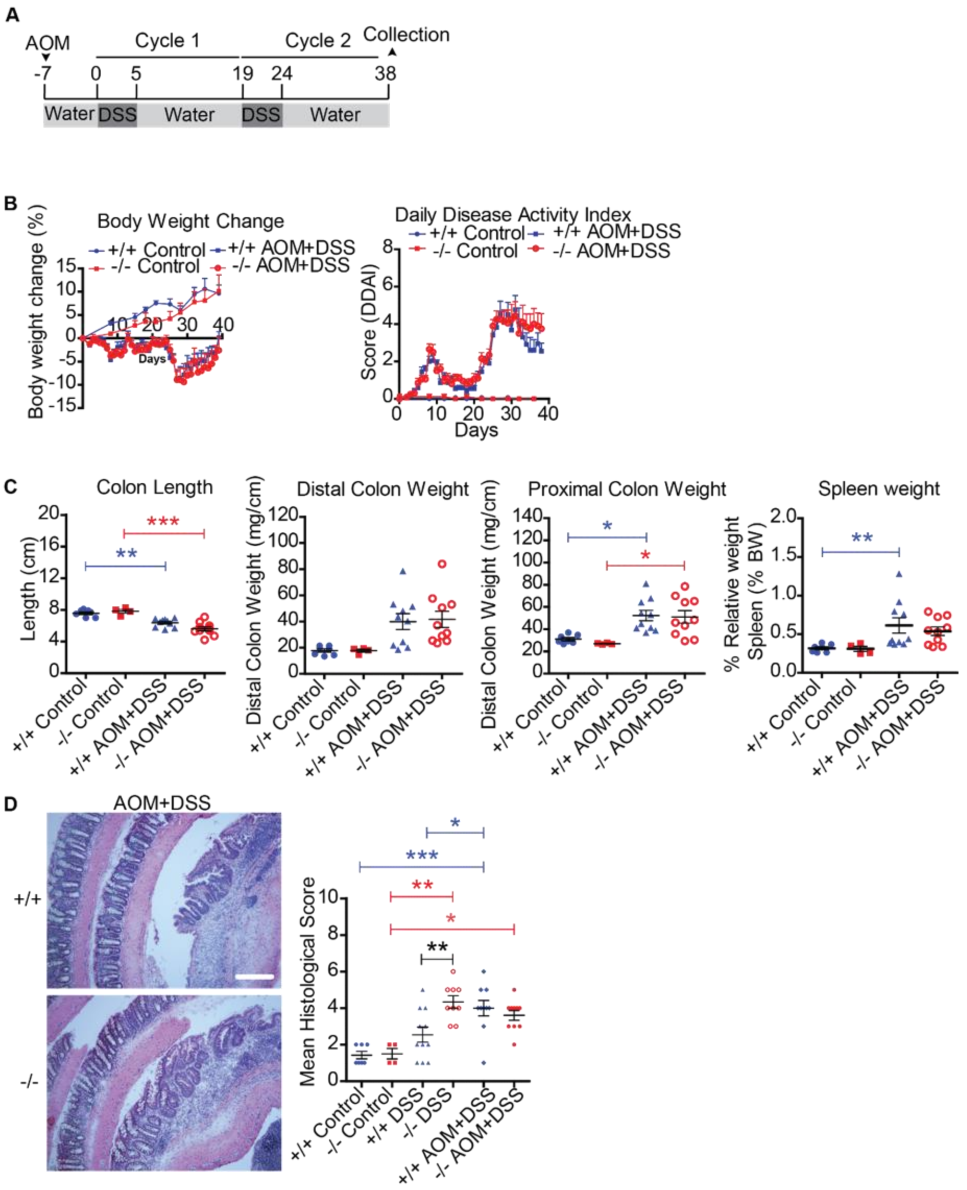

AOM-treated PDLIM2+/+ and PDLIM2-/- mice displayed similar levels of disease severity.

PDLIM2+/+ and PDLIM2-/- mice were pretreated with AOM one week prior to receiving 5 days of DSS followed by 2 weeks of H_2O for a total of two repetitive cycles to initiate the formation of tumours (A). The DDAI and body weight change for the untreated groups and the AOM+DSS group were measured daily over the course of the trial. Each point graphed represents the mean ± SEM per group at each time point and the overall study was stopped on day 38 following two cycles of DSS.

(B). Total colon length, distal colon weight, and proximal colon weight, and spleen weight were measured from untreated and AOM+DSS treated PDLIM2+/+ and PDLIM2 -/- mice (C). Representative H&E staining of histology tissue from PDLIM2+/+ and PDLIM2-/- mice who received AOM+DSS (D). Original images were acquired at 10x magnification with scale bar representing 200µm. Histological scoring was calculated from H&E-stained tissue by assessing the degree of immune infiltration and architectural loss observed. Statistical analysis between groups was calculated by one-way ANOVA with Bonferroni post-hoc analysis. The asterisk (*) represent significant values where * $P < 0.05$, ** $P < 0.01$, and *** $P < 0.001$.

Supplementary Figure 3

Alpha and beta diversity of the gut microbiota in PDLIM2+/+ and PDLIM2-/- mice treated with either PBS or DSS.

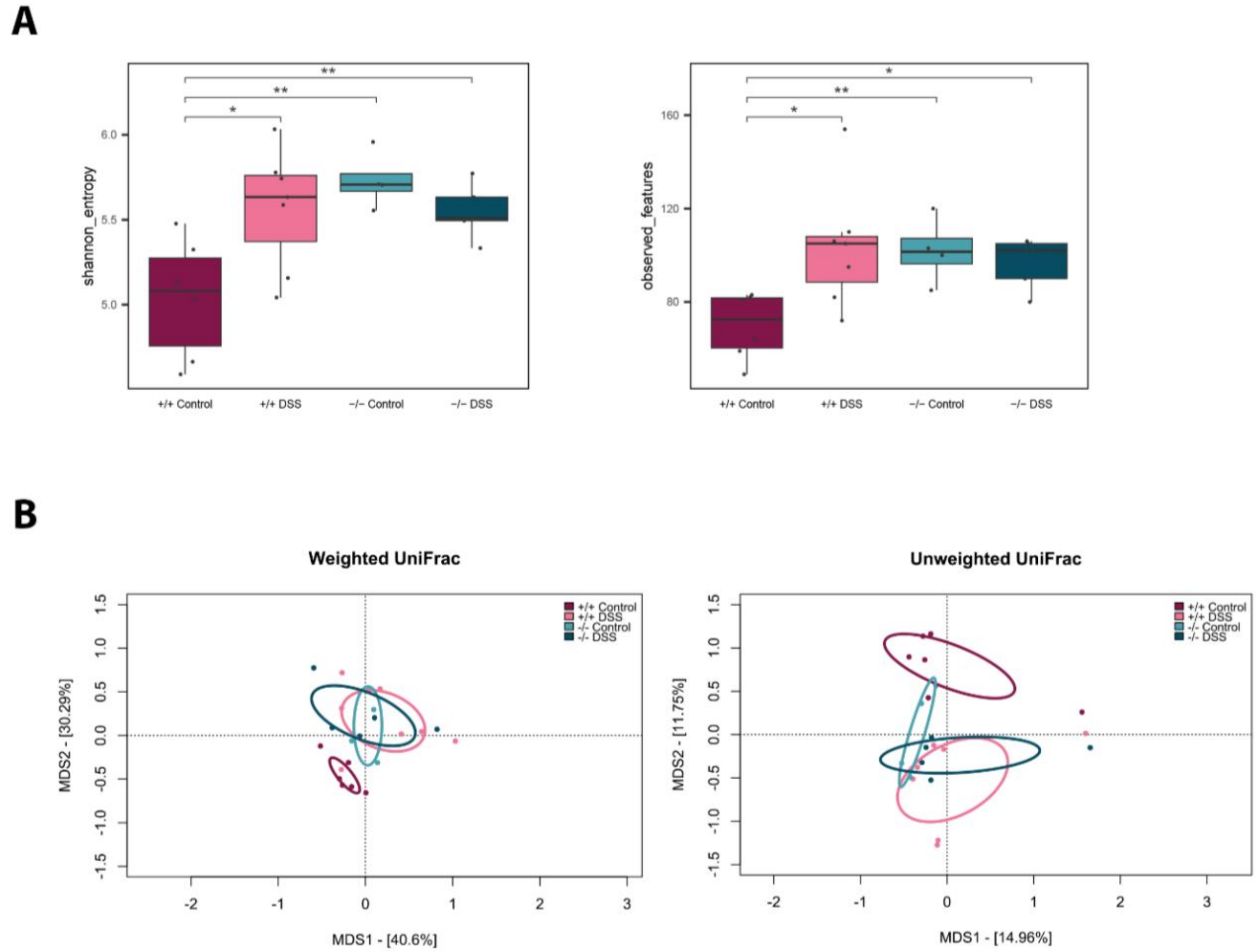

Boxplots showing the distribution of alpha diversity, estimated with Shannon index (left) and the number of observed ASVs (observed_features; right), in faecal samples from PDLIM2+/+ mice treated with PBS ("+/+ Control") or DSS ("+/+ DSS") and PDLIM2-/- mice treated with PBS ("-/- Control") or DSS ("-/- DSS"). Wilcoxon test, * $p \leq 0.05$; ** $p \leq 0.01$ (A). Principal Coordinates Analysis (PCoA) based on weighted UniFrac (left) and unweighted UniFrac (right) distances between the gut microbiota profiles of the study groups. A significant separation was observed between "+/+ Control" samples and all other samples in the weighted UniFrac-based PCoA (PERMANOVA (Adonis), $p<0.006$).

Supplementary Figure 4

Gut microbiota taxa differentially represented in PDLIM2+/+ and PDLIM2-/- mice treated with either PBS or DSS.

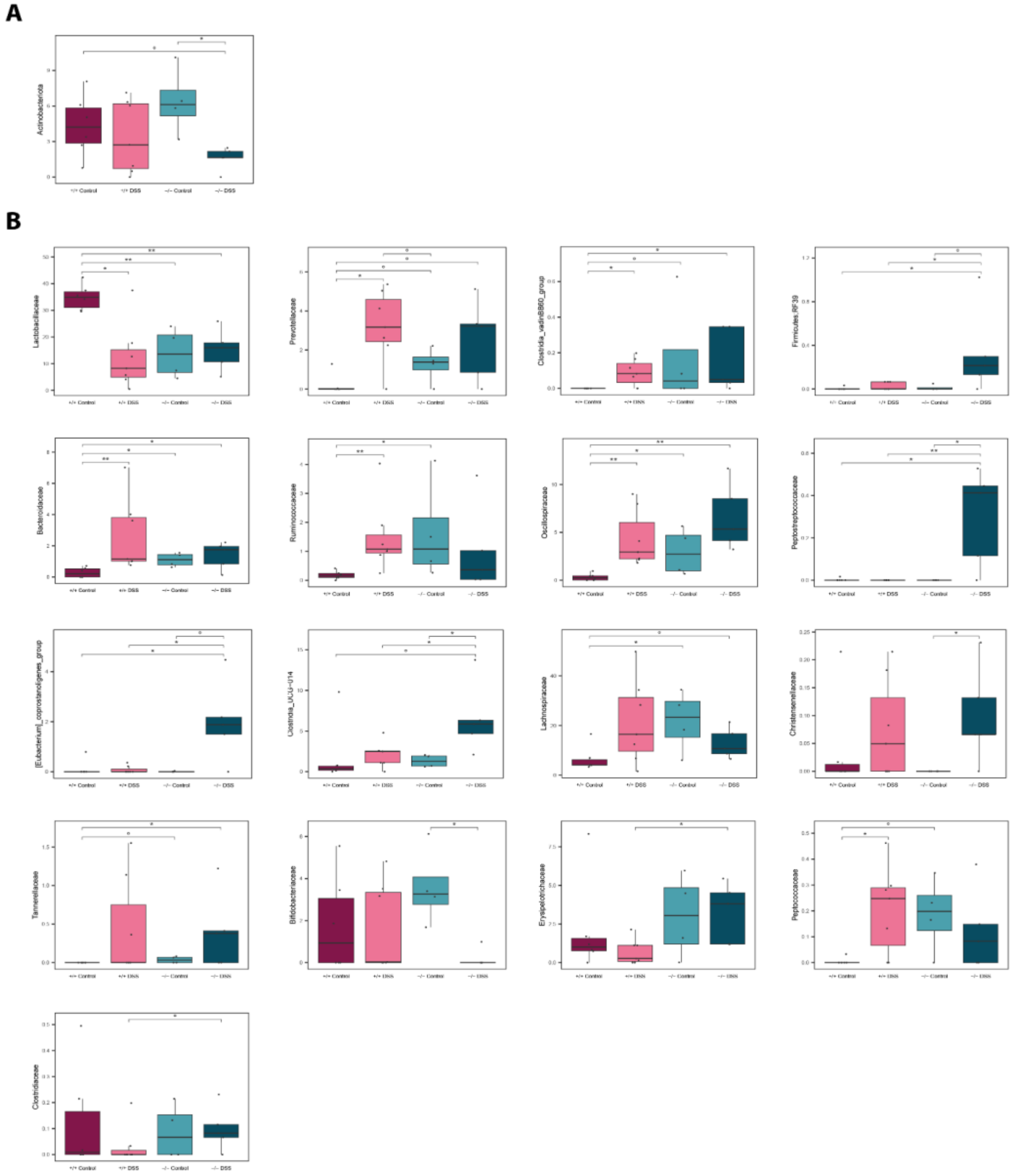

C

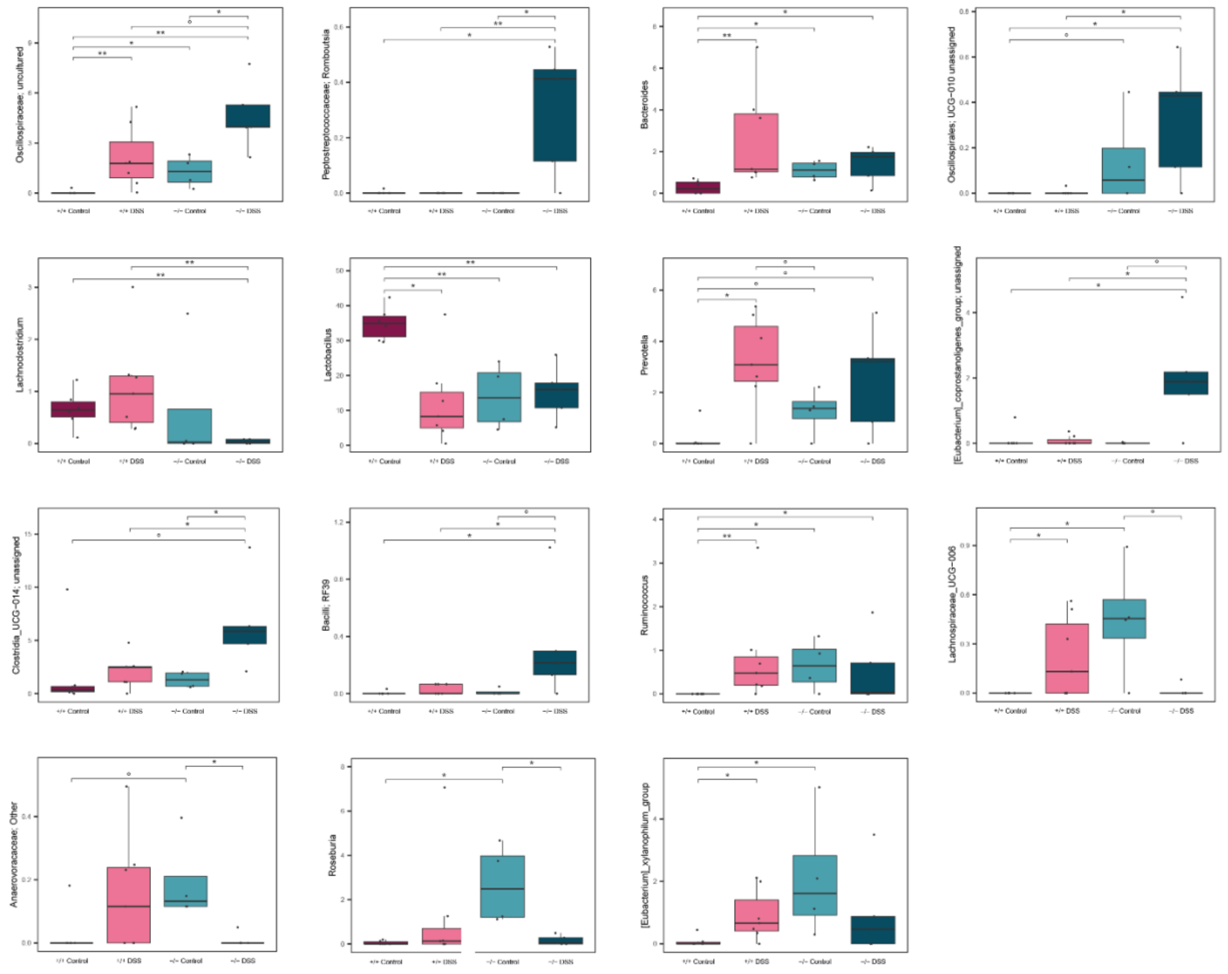

Gut microbiota taxa differentially represented in PDLIM2+/+ and PDLIM2-/- mice treated with either PBS or DSS. Boxplots showing the relative abundance (%) distribution of phyla (A), families (B) and genera (C) differentially represented between PDLIM2+/+ mice treated with PBS ("+/+ Control") or DSS ("+/+ DSS") and PDLIM2-/- mice treated with PBS ("-/- Control") or DSS ("-/- DSS"). Wilcoxon test, * p≤0.05; ** p≤0.01; ° p≤0.09.

Supplementary Figure 5

Discriminating genera of PDLIM2+/+ and PDLIM2-/- mice treated with either PBS or DSS.

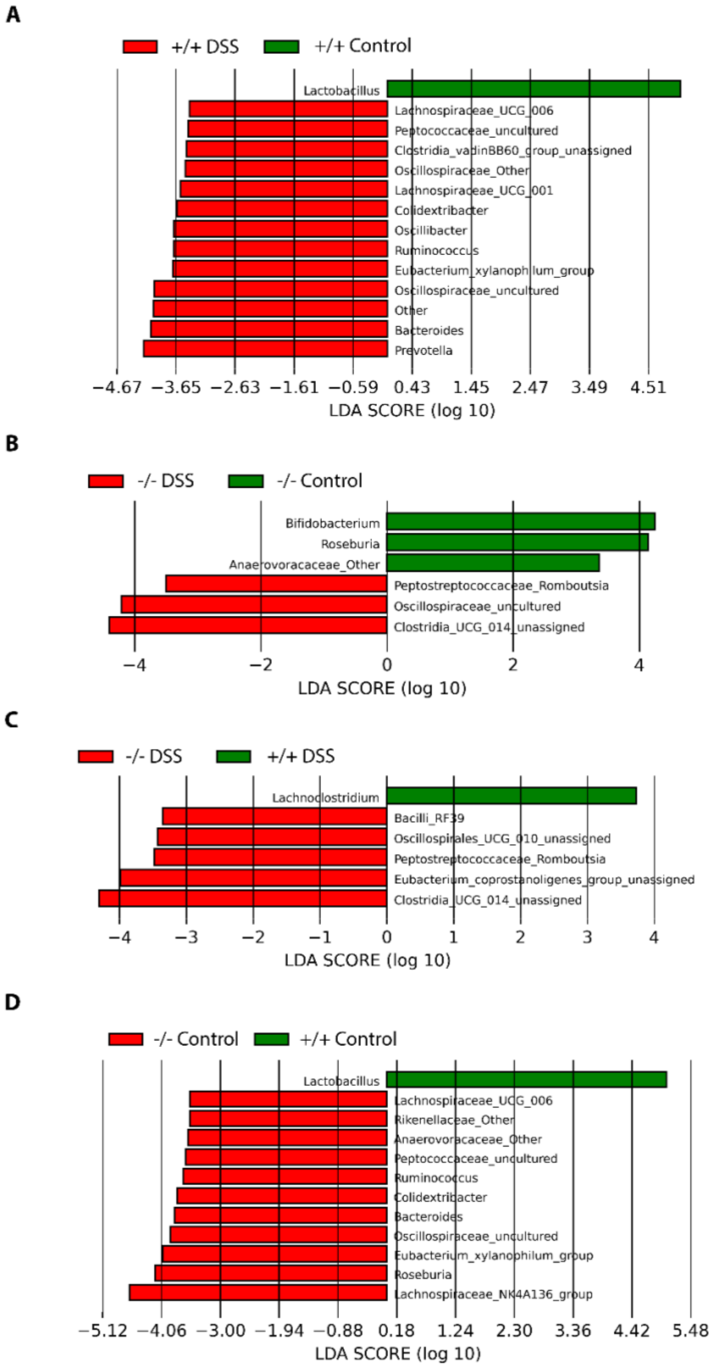

Discriminating genera of PDLIM2+/+ and PDLIM2-/- mice treated with either PBS or DSS. Linear discriminant analysis (LDA) combined with effect size measurements (LEfSe) provided a list of features that enabled discrimination between PDLIM2+/+ mice treated with PBS ("+/+

Control”) and PDLIM2+/+ mice treated with DSS (“+/+ DSS”) (A), PDLIM2-/- mice treated with PBS (“-/- Control”) and PDLIM2-/- mice treated with DSS (“-/- DSS”) (B), “+/+ DSS” vs “-/- DSS” (C), and “+/+ Control” vs “-/- Control” (D). A p-value <0.05 and a score ≥ 2.0 were considered significant.

Supplementary Figure 6:

Predicted KEGG pathways differentially represented in PDLIM2+/+ and PDLIM2-/- mice treated with either PBS or DSS.

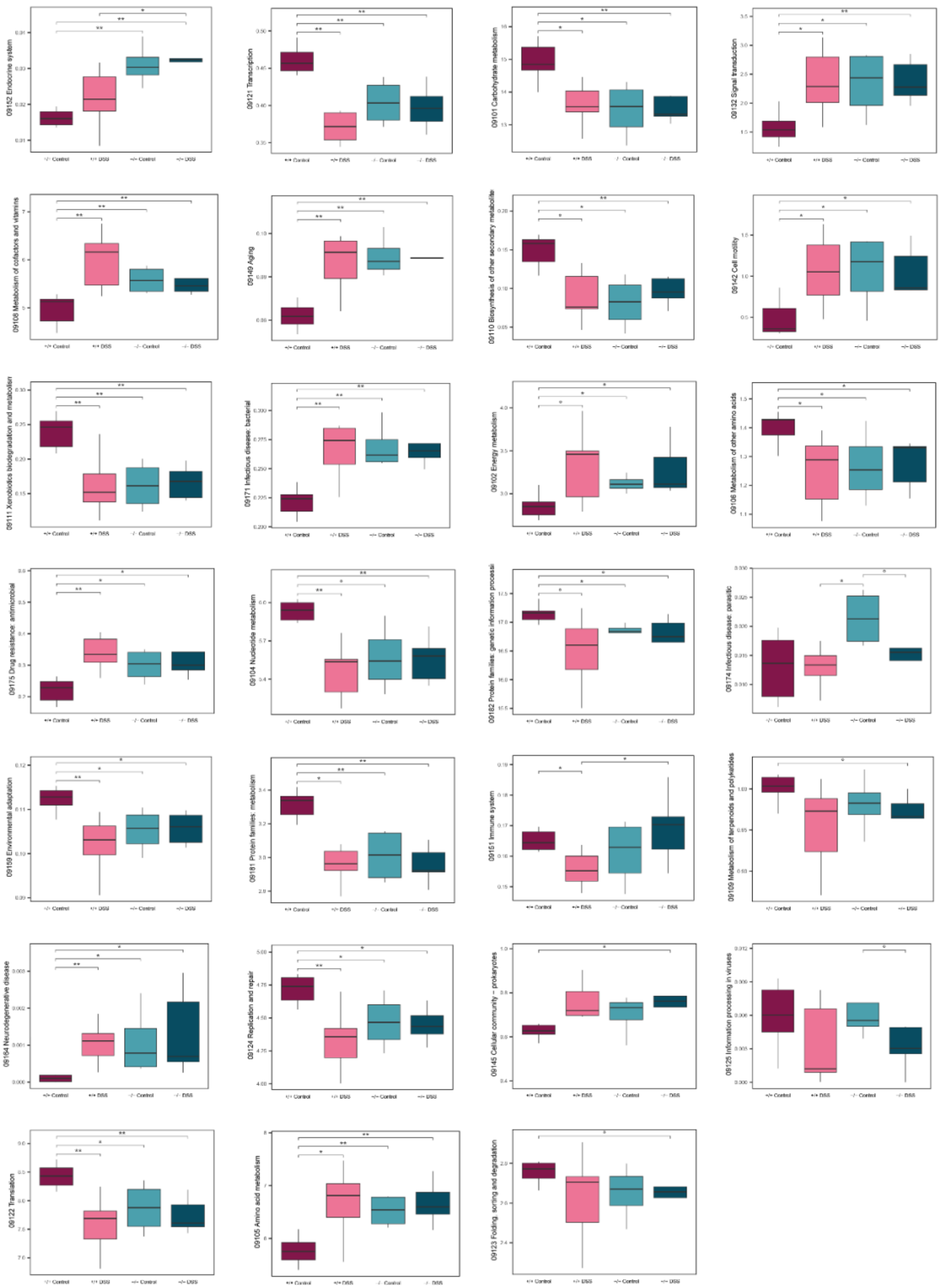

Predicted KEGG pathways differentially represented in PDLIM2+/+ and PDLIM2-/- mice treated with either PBS or DSS. Boxplots showing the relative abundance (%) distribution of KEGG pathways predicted by PICRUSt2 differentially represented between PDLIM2+/+ mice treated with PBS (“+/+ Control”) or DSS (“+/+ DSS”) and PDLIM2-/- mice treated with PBS (“-/- Control”) or DSS (“-/- DSS”). Wilcoxon test, * p≤0.05; ** p≤0.01; ° p≤0.09.

Supplementary Figure 7

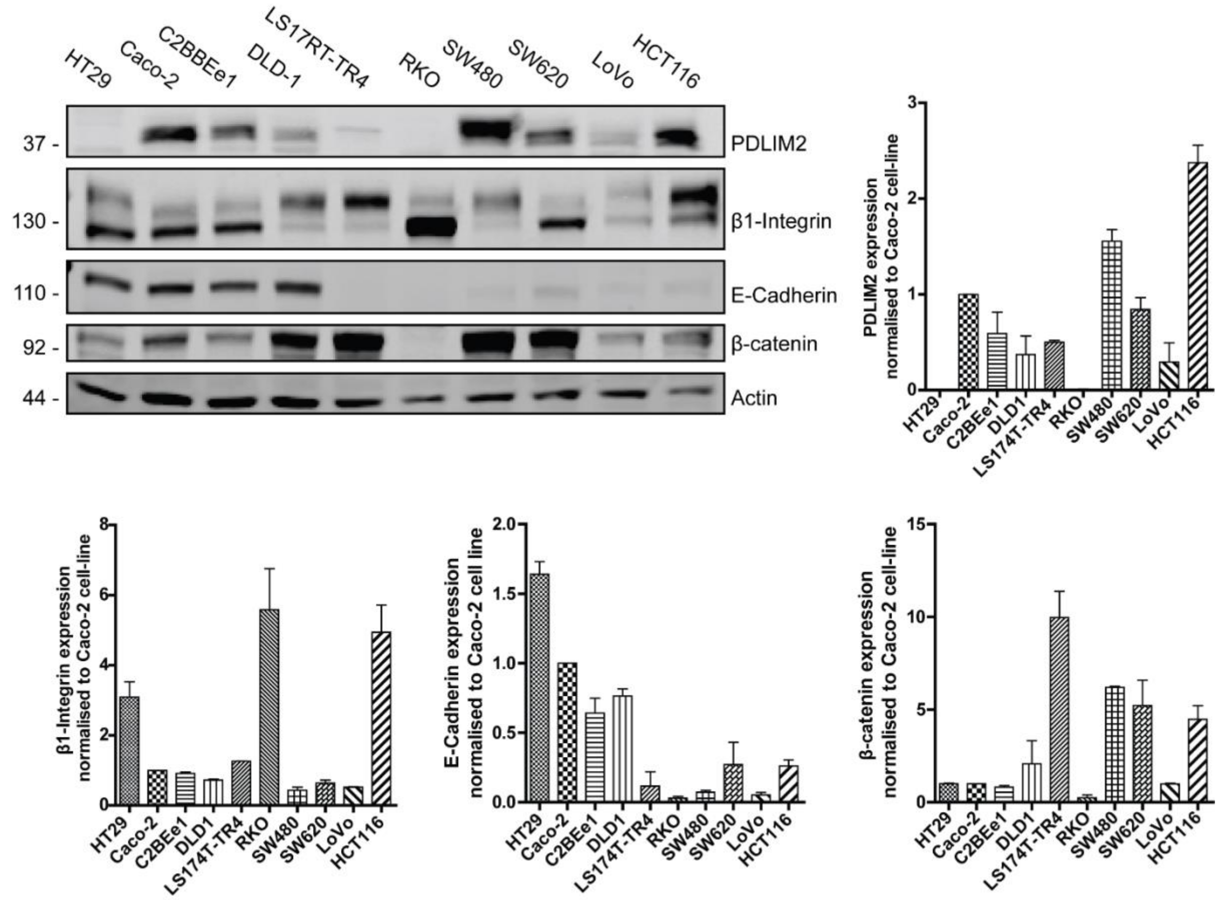

PDLIM2, B1 Integrin, E Cadherin and Beta Catenin are all expressed in Caco- 2 cells.
A panel of 10 CRC epithelial cell lines (HT29, Caco-2, C2BBEe1, DLD-1, LS17RT-TR4, RKO, SW480, SW620, LoVo, and HCT116) were cultured for 48hrs before protein lysis and sample resolution by SDS-PAGE electrophoresis. Blots were probed for the expression of PDLIM2, β1-Integrin, E-Cadherin, β-catenin, and actin. Bar charts represented the average of two independent experiments with expression normalised to the Caco-2 cell-line

Supplementary Figure 8

Caco-2 Cells with PDLIM2 suppressed are impaired in spheroid formation

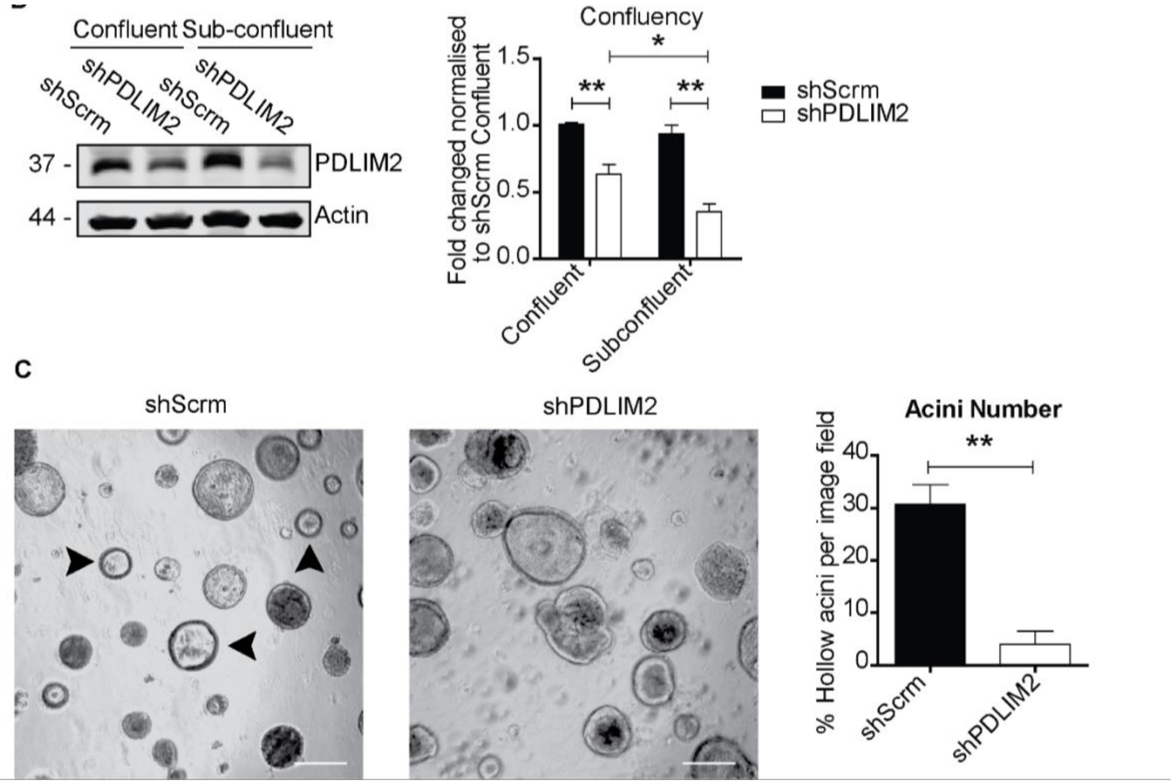

ShScrm and shPDLIM2 Caco-2 cells were seeded as confluent or sub-confluent cultures and assessed for PDLIM2 expression. (C) Cells were cultured for 8 days in complete medium supplemented with 2% Matrigel. On the left is a representative images of spheroid formeation for quantification with scale bars representing 100µm. The percentage of spheroid formed per image field were calculated and arrow heads present spheroid with hollow lumen. Bar plots represent the mean ± SEM of three independent experiments. P-values were calculated using the student's t-test and * = $P < 0.05$, ** = $P < 0.01$, and *** = $P < 0.001$.

Supplementary Table I.

Exact p-values from the Wilcoxon rank-sum tests comparing gut microbiota features across the experimental groups. Pairwise comparisons were performed between PDLIM2 +/+ mice treated with PBS (+/+ Control) or DSS (+/+ DSS) and PDLIM2 −/− mice treated with PBS (−/− Control) or DSS (−/− DSS). The analyses included the relative abundances of phyla and families, as well as the predicted KEGG and MetaCyc pathways. See also Figure 4C,D, Supplementary Figure 3A,B, and Supplementary Figure 5.

	**+/+ Control vs +/+ DSS**	**+/+ Control vs -/- Control**	**+/+ Control vs -/- DSS**	**+/+ DSS vs -/- Control**	**+/+ DSS vs -/- DSS**	**-/- Control vs -/- DSS**
**PHYLUM**						
**Actinobacteriota**			0.051			0.015
**FAMILIES**						
Oscillospiraceae	0.003	0.025	0.008			
Peptostreptococcaceae			0.028		0.009	0.044
Bacteroidaceae	0.003	0.023	0.034			
Lactobacillaceae	0.022	0.009	0.004			
Prevotellaceae	0.015	0.088	0.068	0.058		
Ruminococcaceae	0.006	0.025				
Clostridia_UCG-014			0.052		0.048	0.016
Firmicutes;RF39			0.028		0.042	0.073
[Eubacterium]_coprostanoligenes_group			0.028		0.03	0.073
Clostridia_vadinBB60_group	0.018	0.09	0.015			
Erysipelotrichaceae					0.0145	

Peptococcaceae	0.036	0.055				
Lachnospiraceae		0.038	0.052			
Christensenellaceae						0.043
Bifidobacteriaceae						0.015
Tannerellaceae		0.094	0.048			
Clostridiaceae					0.089	
**KEGG PATHWAYS**						
**09152 Endocrine system**		0.009	0.004		0.01	
**09121 Transcription**	0.005	0.009	0.004			
**09108 Metabolism of cofactors and vitamins**	0.002	0.009	0.008			
**09149 Aging**	0.005	0.009	0.004			
**09111 Xenobiotics biodegradation and metabolism**	0.005	0.009	0.004			
**09171 Infectious disease: bacterial**	0.008	0.009	0.004			
**09175 Drug resistance: antimicrobial**	0.002	0.038	0.017			
**09104 Nucleotide metabolism**	0.002	0.066	0.009			
**09159 Environmental adaptation**	0.002	0.038	0.017			
**09181 Protein families: metabolism**	0.014	0.009	0.004			
**09164 Neurodegenerative disease**	0.005	0.042	0.013			
**09124 Replication and repair**	0.005	0.038	0.017			

**09122 Translation**	0.005	0.038	0.009			
**09105 Amino acid metabolism**	0.014	0.009	0.009			
**09101 Carbohydrate metabolism**	0.022	0.019	0.004			
**09132 Signal transduction**	0.014	0.038	0.009			
**09110 Biosynthesis of other secondary metabolites**	0.073	0.019	0.004			
**09142 Cell motility**	0.014	0.038	0.03			
**09102 Energy metabolism**	0.051	0.038	0.017			
**09106 Metabolism of other amino acids**	0.022	0.067	0.03			
**09182 Protein families: genetic information processing**	0.051	0.019	0.052			
**09174 Infectious disease: parasitic**				0.012		0.063
**09151 Immune system**	0.035				0.048	
**09109 Metabolism of terpenoids and polyketides**			0.052			
**09145 Cellular community - prokaryotes**			0.03			
**09125 Information processing in viruses**						0.063
**09123 Folding, sorting and degradation**			0.082			
**METACYC PATHWAYS**						
**RHAMCAT-PWY**	0.001	0.067	0.004	0.042		
**LACTOSECAT-PWY**	0.022	0.009	0.004			

**P122-PWY**	0.005	0.019	0.004			
**P124-PWY**	0.014	0.009	0.004			
**GALACT-GLUCUROCAT-PWY**	0.035		0.03			
**GLUCUROCAT-PWY**	0.022		0.03	0.024		
**NONOXIPENT-PWY**			0.052			
**PANTOSYN-PWY**	0.035					
**FAO-PWY**			0.009		0.03	0.063
