# Supplementary material for: Loss of the PDLIM2 protein during chronic colitis promotes inflammation, impaired epithelium recovery, alterations to the microbiome and oxidative stress
Source: Front Endocrinol (Lausanne). 2026 Feb 10;16:1720162. doi: 10.3389/fendo.2025.1720162 (PMC12929155; doi:10.3389/fendo.2025.1720162)

Full length Images for western blots shown in Figures 5 and 6

Panel A

Rb B1 Integrin   Lanes 1 and 2

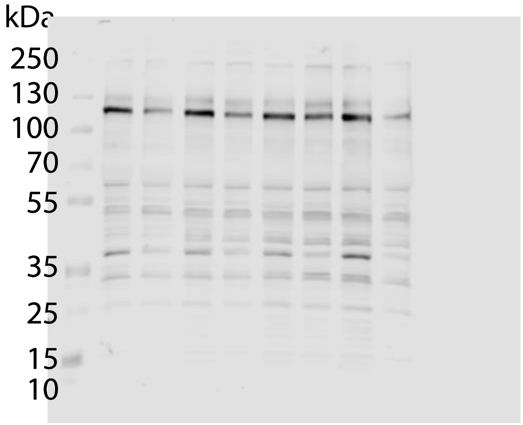

Rb B3 Integrin Lane 1 and 2

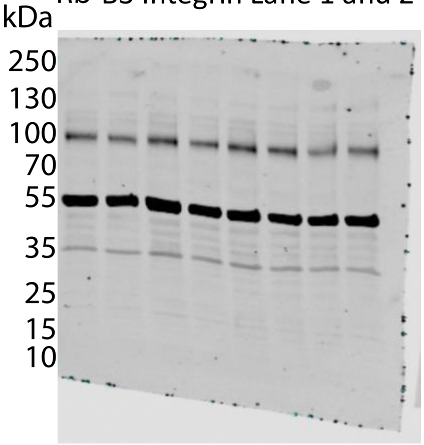

Rb Beta-catenin Lanes 1 and 2

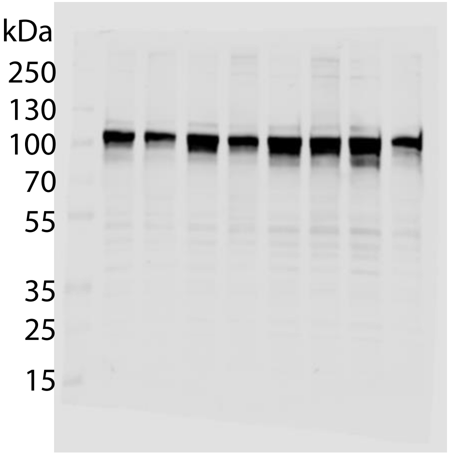

Ms E-cadherin Lanes 1 and 2

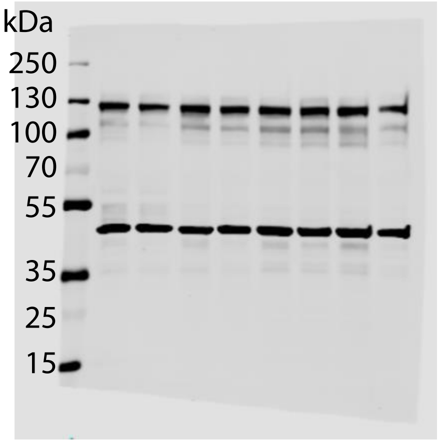

Ms PDLIM2 Lanes 1 and 2

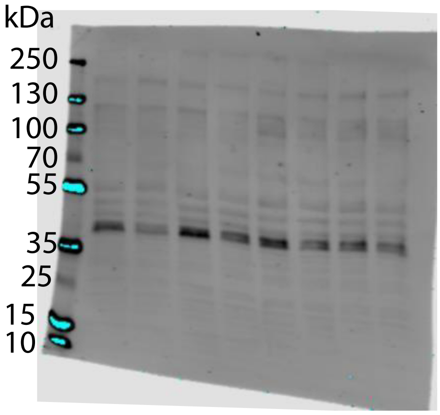

Ms Actin Lanes 1 and 2

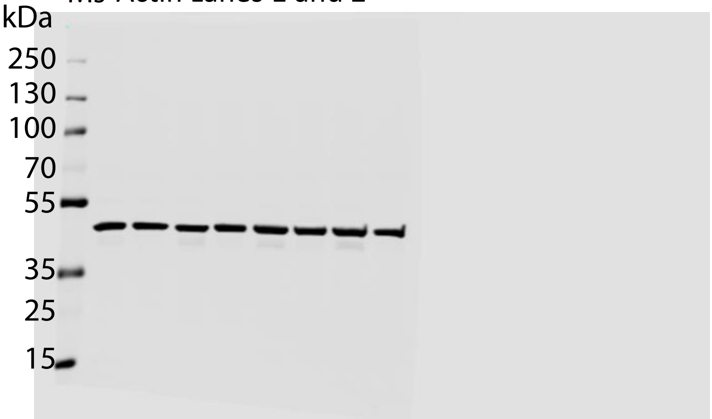

Figure 5 Panel C

B1 Integrin

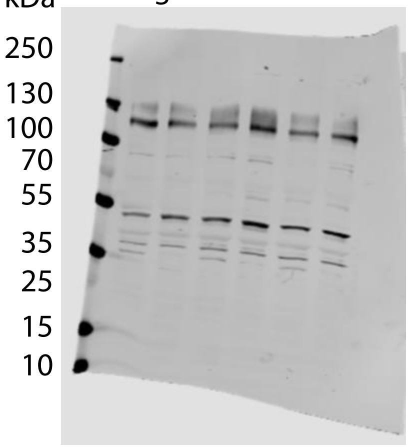

PDLIM2

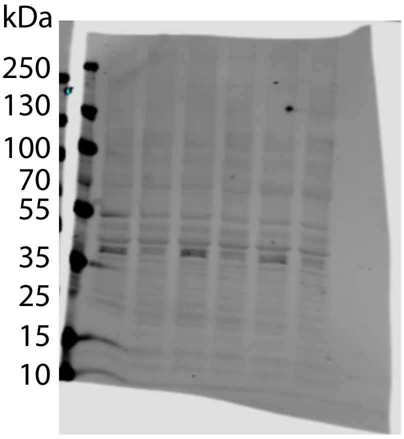

Rb GAPDH

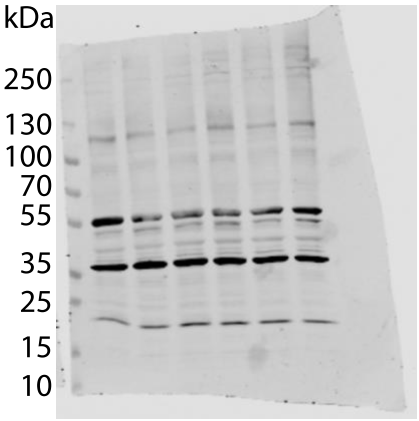

Figure 6
Panel A (left blot)
PDlim2 and Actin

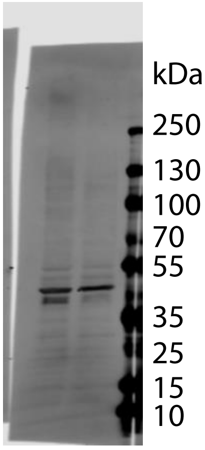

Figure 6 Panel A (right blot)
Rb Glut 1 (lanes 3 and 4)

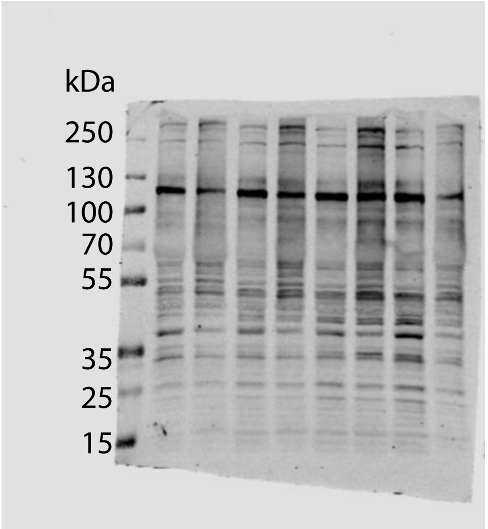

Ms PDLIM2 Lanes 3 and 4

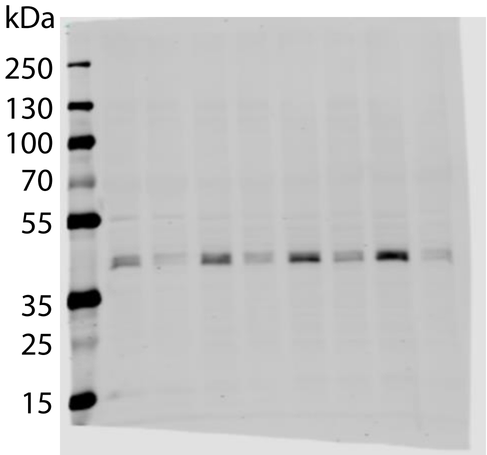

Actin Lanes 3 and 4

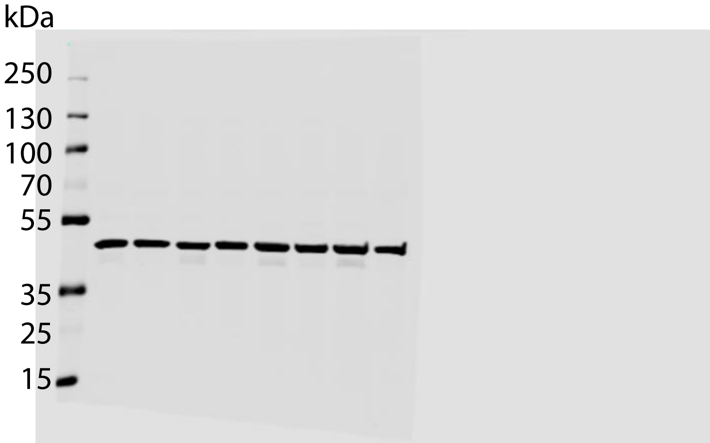

Figure 6 Panel C Lanes 1-4

RbP-AMPK

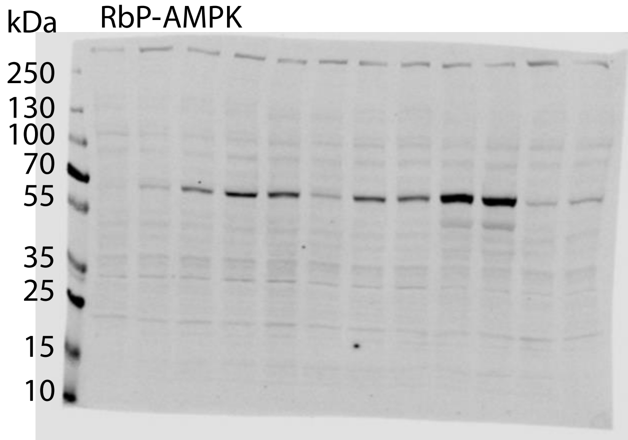

Rb AMPK

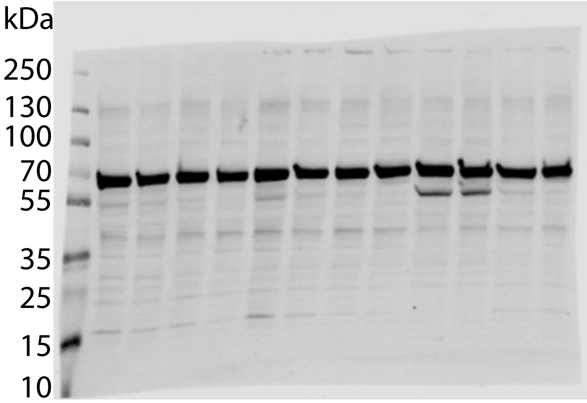

Ms Tubulin blot 1 lanes 1-4

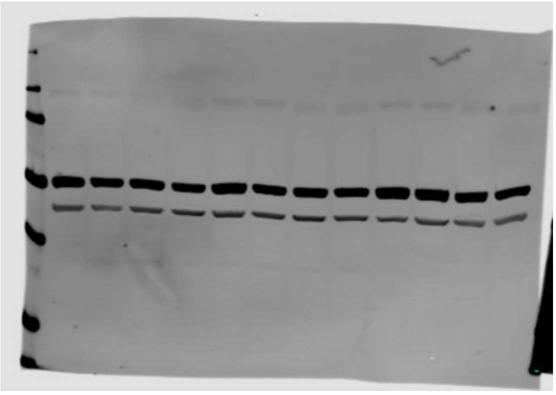

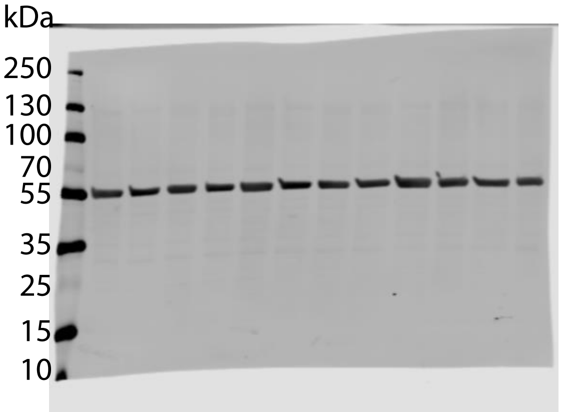

Panel F

Ms PDLIM2 lanes 1 and 2

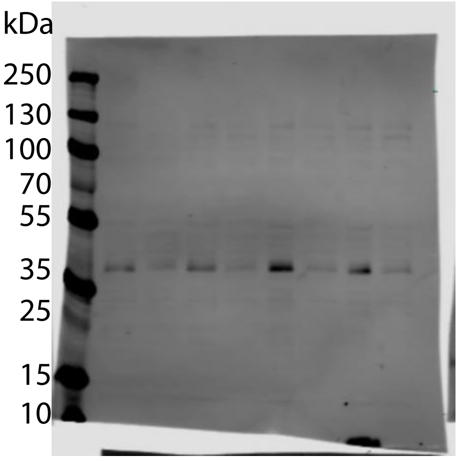

Rb Catalase lanes 2 and 3

Middle bands

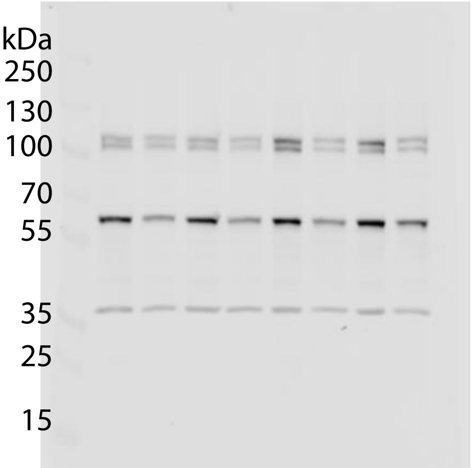

Rb SOD-2 lanes 1 and 2 (second band from bottom)

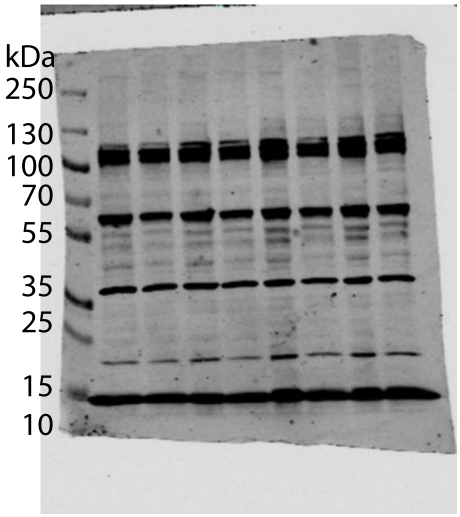

Rb Tom20

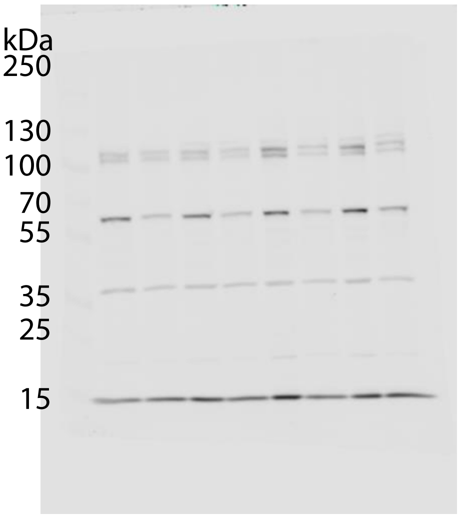

Ms Actin

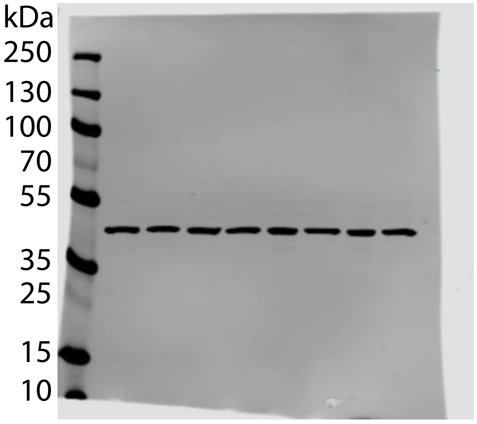

Ms Actin lanes 1 and 2

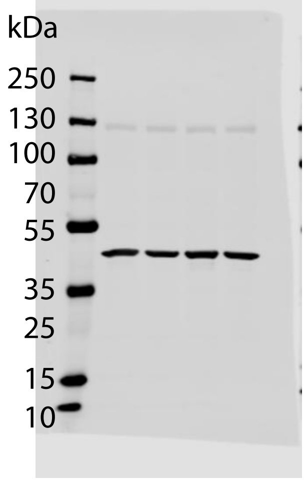

Rb HO-1 lanes 1 and 2

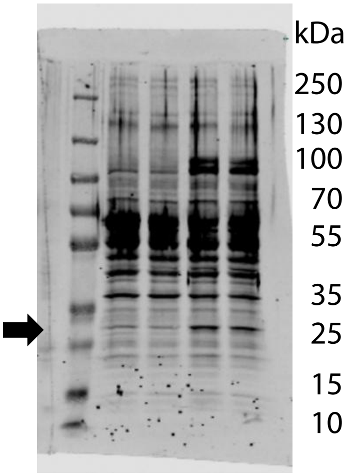

Ms PDLIM2 lanes 1 and 2

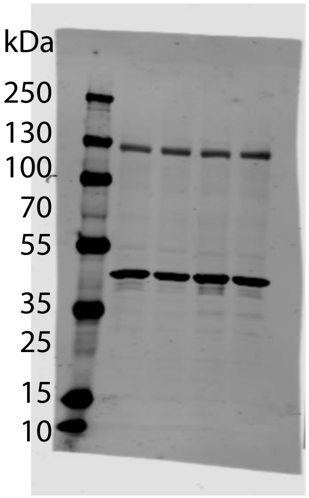

Figure 6 Panel G

Ms PDLIM2 (left side of MW ladder)

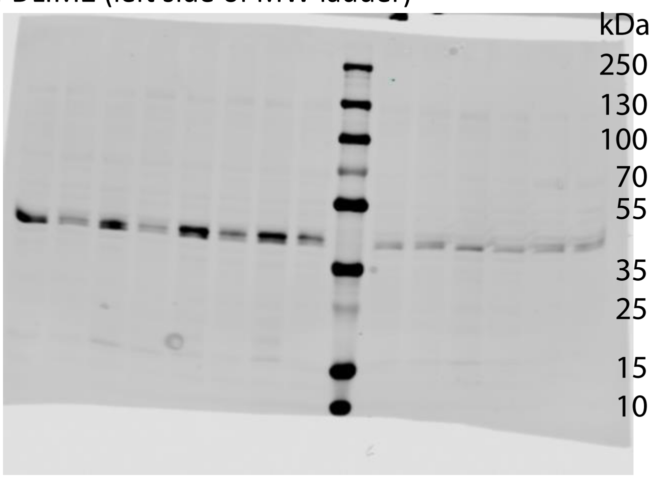

Rb NRF-2 left side of ladder

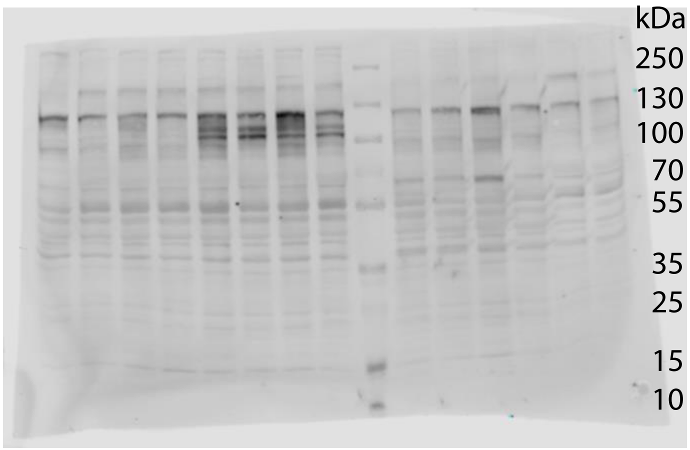

Rb P-FAK upper bands left of ladder)

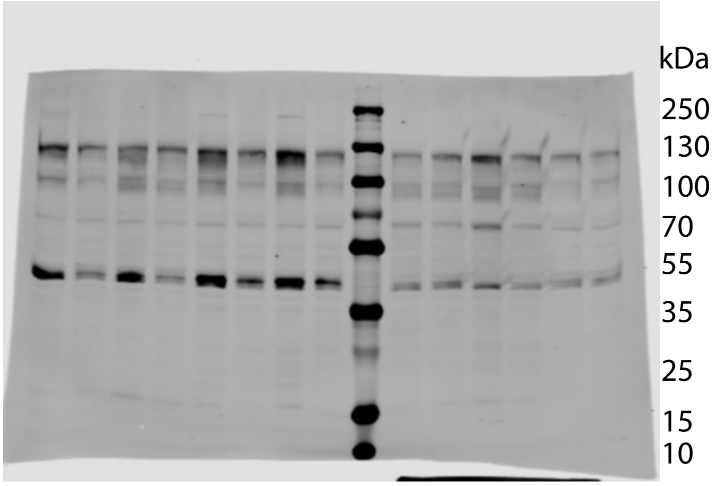

Rat FAK

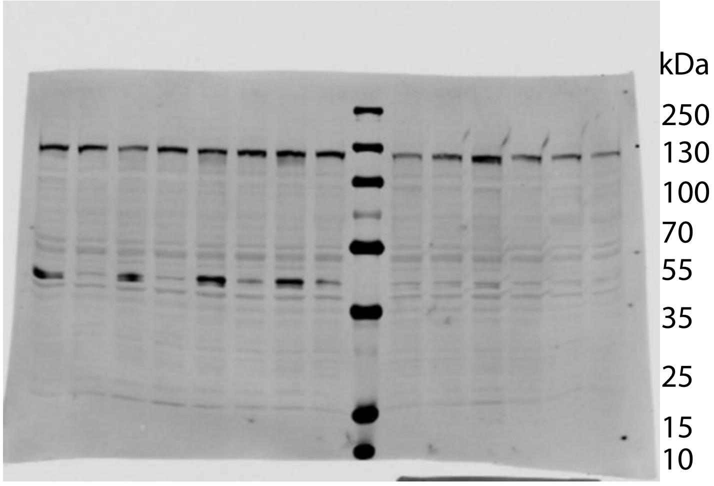

Ms Tubulin

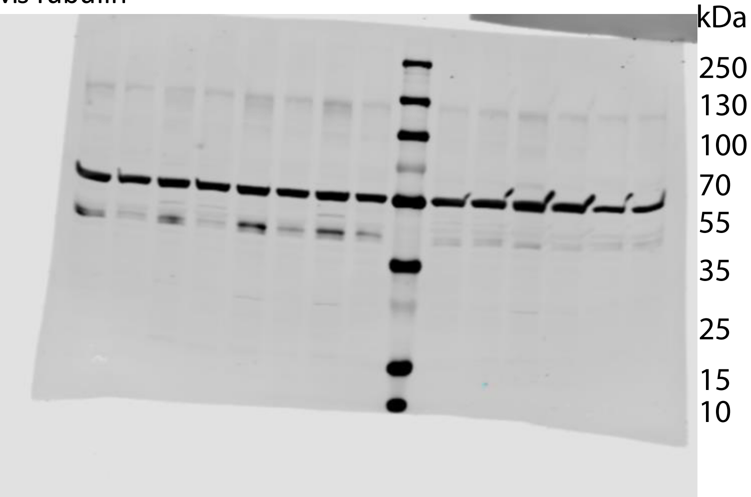

Supplement: Supplementary file 2 [file DataSheet2.pdf]
